# Meta-analyses of QTL for grain yield and anthesis silking interval in 18 maize populations evaluated under water-stressed and well-watered environments

**DOI:** 10.1186/1471-2164-14-313

**Published:** 2013-05-10

**Authors:** Kassa Semagn, Yoseph Beyene, Marilyn L Warburton, Amsal Tarekegne, Stephen Mugo, Barbara Meisel, Pierre Sehabiague, Boddupalli M Prasanna

**Affiliations:** 1 International Maize and Wheat Improvement Center (CIMMYT), P. O. Box 1041, Village Market 00621, Nairobi, Kenya; 2 United States Department of Agriculture-Agricultural Research Service: Corn Host Plant Resistance Research Unit, Box 9555, Mississippi State, MS, 39762, USA; 3 CIMMYT, 12.5 Km peg Mazowe Road, P.O. Box MP163, Mount Pleasant, Harare, Zimbabwe; 4 Monsanto Company, Vermeulen Street, Petit 1512, Gauteng, South Africa; 5 Monsanto SAS, Croix de Pardies, BP21, Peyrehorade, 40310, France

**Keywords:** Breeding, Drought, Heritability, Maize, Managed water stress, Meta analysis, SNP

## Abstract

**Background:**

Identification of QTL with large phenotypic effects conserved across genetic backgrounds and environments is one of the prerequisites for crop improvement using marker assisted selection (MAS). The objectives of this study were to identify meta-QTL (mQTL) for grain yield (GY) and anthesis silking interval (ASI) across 18 bi-parental maize populations evaluated in the same conditions across 2-4 managed water stressed and 3-4 well watered environments.

**Results:**

The meta-analyses identified 68 mQTL (9 QTL specific to ASI, 15 specific to GY, and 44 for both GY and ASI). Mean phenotypic variance explained by each mQTL varied from 1.2 to 13.1% and the overall average was 6.5%. Few QTL were detected under both environmental treatments and/or multiple (>4 populations) genetic backgrounds. The number and 95% genetic and physical confidence intervals of the mQTL were highly reduced compared to the QTL identified in the original studies. Each physical interval of the mQTL consisted of 5 to 926 candidate genes.

**Conclusions:**

Meta-analyses reduced the number of QTL by 68% and narrowed the confidence intervals up to 12-fold. At least the 4 mQTL (mQTL2.2, mQTL6.1, mQTL7.5 and mQTL9.2) associated with GY under both water-stressed and well-watered environments and detected up to 6 populations may be considered for fine mapping and validation to confirm effects in different genetic backgrounds and pyramid them into new drought resistant breeding lines. This is the first extensive report on meta-analysis of data from over 3100 individuals genotyped using the same SNP platform and evaluated in the same conditions across a wide range of managed water-stressed and well-watered environments.

## Background

Globally, maize (*Zea mays* ssp. *mays* L.) is an important source of food and nutritional security for millions of people in the developing world, especially in sub-Saharan Africa (SSA) and Latin America [1]. Maize is a staple food in many of the SSA countries and is commonly grown by resource poor, small-scale farmers in rural areas. It covers 25 million hectares in SSA that produce 38 million metric tons [1] but the average maize yield in the region is estimated at 1.4 tons per hectare, which is about 20%, 37% and 56% of the average maize yield in developed countries, Brazil and Philippines, respectively [2]. Several factors, including high frequency of drought stress, scarcity and high cost of irrigation, and farmers’ inability to obtain quality seeds and fertilizers, contribute to such low productivity in the region. Given the unpredictable nature of drought and climate variability over years, breeders must develop improved maize hybrids that are able to withstand drought stress without significant yield penalty under optimal rainfall conditions [3-5]. For developing drought tolerant maize, selection can be done directly under water stress, indirectly under well-watered (optimal) conditions, or under both optimal and stress conditions [6]. However, heritability of grain yield under water stress has been reported to be lower than yield under optimal environments [7]. Hence, physiologists and breeders have devoted significant efforts in identifying relevant secondary traits correlated to grain yield for indirect selection. These include anthesis silking interval (ASI) between male and female flowering and several other morpho-physiological traits [8,9].

The ability to transfer target genomic regions associated with trait(s) of interest using molecular markers resulted in extensive QTL mapping experiments in most economically important crops. Such studies aimed at the identification of molecular markers for marker assisted backcrossing (MABC), marker assisted recurrent selection (MARS) and QTL cloning [10]. Using MABC, Ribaut and Ragot [4] introgressed 5 QTL associated with yield components and flowering in maize from a donor parent into a drought susceptible recurrent parent. The authors reported increased grain yield and reduced ASI under water-limited conditions. The best MABC progeny outperformed the recurrent parent by two to four times under severe drought conditions, with no yield reduction under optimal conditions. However, drought is a complex trait influenced by genetic background and other environmental factors; thus, relying on a few QTL for MABC is unlikely to create optimally drought tolerant lines for target population of environments. Individual drought associated QTL generally explain a very small proportion of the phenotypic variance for grain yield, ASI or barrenness. QTL for drought related traits are also often cross-specific and remain undetected in crosses from different genetic backgrounds. Most QTL are detected under either drought stress or optimal conditions (not both), and there is no assurance that QTL detected from inbred lines will function in the same manner in hybrids. Thus, they must be fully validated in several environmental conditions and hybrid combinations before deployment in a large breeding program.

MARS is another marker based breeding technology that seeks to accumulate favorable alleles from several genomic regions within a single population [11]. In maize, the MARS protocol involves (a) development and evaluation of testcross performance of bi-parental populations in multi-location experiments; (b) genotyping of the F_2:3_ population (Cycle 0); (c) undertaking an *ad hoc* significance test to identify a subset of markers that are significantly associated with the target trait; and (d) one generation (cycle) of selection of the best Cycle 0 families based on phenotypic index derived from testcross performance, followed by 2-3 cycles of selection based solely on markers with significant effects [12-15]. Currently, the International Maize and Wheat Improvement Center (CIMMYT), in collaboration with the national agricultural research systems (NARS) from 14 countries in Africa, the International Institute of Tropical Agriculture (IITA), the African Agricultural Technology Foundation (AATF), the Monsanto Company, and several regional and national seed companies in Africa, is working in large scale projects that aim to develop and disseminate drought tolerant maize for SSA using conventional breeding, MARS, and/or transgenic technology. These include the drought tolerant maize for Africa (DTMA) and the water efficient maize for Africa (WEMA) projects. For the MARS component of the WEMA project, CIMMYT developed and evaluated 18 bi-parental mapping populations, which formed the base for this study. All these populations have been phenotyped with common protocols and genotyped under a common single nucleotide polymorphism (SNP) platform.

Comparisons among independent QTL mapping projects usually attempt to determine if loci identified in each are the same by comparing the chromosomal position of a common subset of markers across different studies and/or indirectly by comparing each mapping population to a reference map [16]. Co-localized QTL may not be identical, however, especially when they are associated with large confidence intervals. Meta QTL analysis [17] is a better method for combining data from independent studies to detect consensus QTL and to shrink the QTL confidence intervals. Meta-analyses have been used in maize, wheat, rice, rapeseed, potato, cotton, soybean, barley, cocoa and apricot [18,19]. In maize, meta QTL (mQTL) for drought tolerance [20], flowering time [21], grain yield components [22], ear rot resistance [23,24] and silage quality [19] have been reported. Hao and colleagues [20] collected published QTL results and data related to drought tolerance for 12 mapping populations from the MaizeGDB website (http://www.maizegdb.org) and conducted meta-analyses on a total of 239 and 160 QTL detected under water stressed and well watered conditions, respectively. The authors reported 39 consensus mQTL for drought-tolerance related traits under water stress and 36 mQTL under well watered conditions. In most QTL meta-analyses published so far [19,20,23,24], authors compiled published linkage maps and QTL results from independent studies using different phenotyping protocols, constructed consensus linkage maps using a subset of markers common to the different studies, and projected mQTL positions and their confidence intervals onto the consensus map. Limitations of those studies are caused by the use of different phenotyping protocols, different QTL mapping methods, too few common markers, or by too few populations, causing lower confidence in the mQTL and the delimited intervals. The objectives of the present study were to identify mQTL for grain yield and ASI across 18 bi-parental maize populations genotyped with a common SNP platform and phenotyped with a common protocol in multi-location experiments both under water stressed and well watered environments.

## Results

### Phenotypic distribution, heritability and correlations

The mean phenotypic distribution for GY and ASI for all 18 bi-parental populations under water stressed and optimum environments was either normal or approximately normal (data not shown). Broad-sense heritability for GY (Table 1) varied from 0 to 0.40 under water stressed and from 0.23 to 0.58 under optimum environments. For ASI, heritability under water-stressed and optimum environments varied from 0 to 0.37 and from 0.08 to 0.54, respectively (Table 2). Populations with heritability < 0.1 under stressed (6 populations for each trait, of which 2 were common between the two traits) and/or < 0.20 under optimum (only 2 pops for ASI) environments were excluded from QTL analysis. In the dataset used for QTL mapping, therefore, broad sense heritability for GY and ASI varied from 0.13 to 0.40 under water stressed and from 0.21 to 0.58 under optimum conditions. There was significant but low to moderate negative correlation between GY and ASI under stressed (-0.09 to -0.51; p <0.001) and optimum (-0.08 to -0.23; p <0.001) conditions (data not shown).

**Table 1:** Summary of the QTL for grain yield detected in 18 bi-parental populations

**Population code**	**Water stressed**		**Well watered**		**Heritability**		Genetic variance per QTL**	
	**No. of QTL**	**R**^**2 **^(%)*	**No. of QTL**	**R**^**2 **^(%)*	**Water stressed**	**Well watered**	**Water stressed (%)**	**Well watered (%)**
6x1008	2	6.10	4	21.60	0.24	0.42	12.76	12.92
6x1015	0	0.00	7	42.70	0.26	0.35	0.00	17.48
6x1016	-	-	5	37.90	0.00	0.57	0.00	13.37
6x1017	-	-	7	65.70	0.00	0.47	0.00	19.97
6x1018	1	7.70	5	34.00	0.26	0.52	29.96	13.13
6x1019	-	-	6	35.20	0.00	0.38	0.00	15.60
6x1020	2	6.10	5	24.00	0.33	0.55	9.27	8.66
6x1021	9	48.80	7	71.70	0.21	0.58	25.34	17.69
6x1023	-	-	3	28.50	0.08	0.38	0.00	25.13
6x1024	-	-	5	43.70	0.02	0.44	0.00	19.73
6x1028	-	-	7	50.00	0.00	0.40	0.00	17.86
6x1115	0	0.00	3	11.10	0.22	0.29	0.00	12.76
6x1116	3	9.30	5	62.40	0.24	0.49	12.92	25.47
6x1117	0	0.00	2	13.70	0.27	0.48	0.00	14.27
6x1118	0	0.00	2	11.70	0.40	0.26	0.00	22.50
6x1120	0	0.00	3	26.30	0.11	0.51	0.00	17.19
6x1121	1	3.90	6	60.60	0.32	0.23	12.19	43.91
6x1122	0	0.00	1	1.50	0.36	0.37	0.00	4.05
Total	18.00	81.90	83.00	642.30	3.32	7.68	102.43	321.69
Mean	1.50	6.83	4.61	35.68	0.18	0.43	5.69	17.87

*R^2^ = the total phenotypic variance explained by all QTL.

** The genetic variance is the percent phenotypic variance explained by a single QTL divided by heritability of a trait.

Maize populations and environments are described in detail in Table 3.

**Table 2:** Summary of the QTL for ASI detected in bi-parental populations

**Population code**	**Water stressed**		**Well watered**		**Heritability**		Genetic variance per QTL**	
	**No. of QTL**	**R**^**2 **^(%)*	**No. of QTL**	**R**^**2 **^(%)*	**Water stressed**	**Well watered**	**Water stressed**	**Well watered**
6x1008	3	13.40	3	21.80	0.27	0.40	16.54	18.17
6x1015	-	-	4	26.10	0.00	0.21	0.00	31.07
6x1016	1	1.80	1	4.70	0.13	0.36	13.85	13.06
6x1017	1	8.20	1	12.30	0.19	0.41	43.16	30.00
6x1018	-	-	-	-	0.00	0.08	0.00	0.00
6x1019	0	0.00	2	14.40	0.03	0.33	0.00	21.82
6x1020	5	30.60	4	28.40	0.26	0.33	23.54	21.52
6x1021	2	5.40	4	17.20	0.20	0.34	13.50	12.65
6x1023	3	18.20	5	44.30	0.29	0.45	20.92	19.69
6x1024	-	-	5	19.80	0.05	0.51	0.00	7.76
6x1028	6	23.20	5	43.20	0.23	0.37	16.81	23.35
6x1115	-	-	-	-	-	-	-	-
6x1116	-	-	7	38.70	0.00	0.46	0.00	12.02
6x1117	3	16.70	0	0.00	0.14	0.24	39.76	0.00
6x1118	-	-	0	0.00	0.00	0.23	0.00	0.00
6x1120	3	17.20	3	17.10	0.21	0.47	27.30	12.13
6x1121	2	12.90	0	0.00	0.37	0.54	17.43	0.00
6x1122	4	16.60	5	40.80	0.24	0.42	17.29	19.43
Total	33.00	164.20	49.00	328.80	2.61	6.15	250.10	242.65
Mean	2.75	13.68	3.06	20.55	0.15	0.36	14.71	14.27

*R^2^ = the total phenotypic variance explained by all QTL.

** The genetic variance is the percent phenotypic variance explained by a single QTL divided by heritability of a trait.

Maize populations and environments are described in detail in Table 3.

### Linkage and consensus mapping

**Figure 1:**
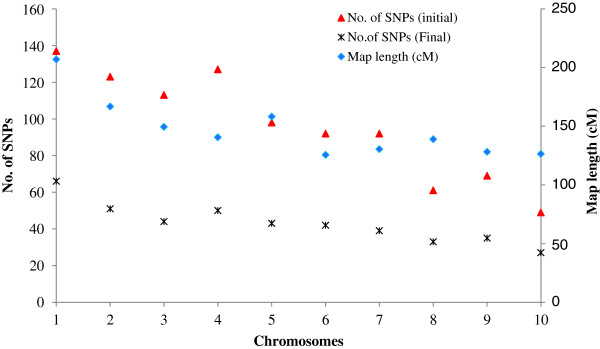
Summary of the consensus map of 10 maize chromosomes, showing map length, initial number of SNPs integrated in the consensus maps and final number of SNPs retained for meta analyses after excluding all markers with map distance of <1 cM.

**Figure 2:**
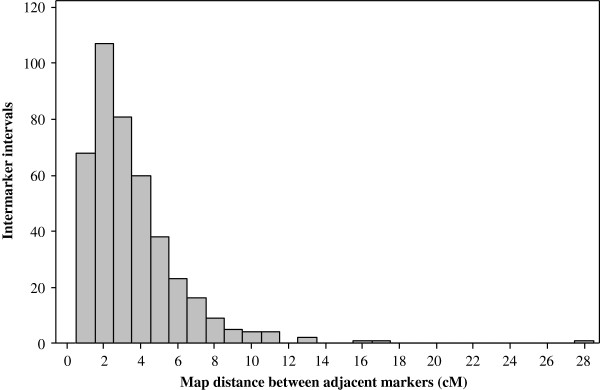
Frequency distribution of the 430 SNPs that were used for generating the final consensus linkage maps.

The map length in the population specific linkage maps varied from 426 to 1418 cM, with a mean of 1075 cM (Table 3). The total number of mapped SNPs per population varied from 118 to 202 with an average of 172.3. The average number of SNPs mapped per chromosome in the population specific maps was 17.1 (data not shown). Chromosome 10 had fewer markers (range 4-13; average 9) compared to all other chromosomes due to low marker polymorphism in the initial polymorphism screening between parents (Figure 1). The mean map distance between markers ranged from 2.8 to 7.8 cM and the overall mean across all 18 populations was 6.1 cM. The final consensus map consisted of 430 SNPs with a total map length of 1471 cM. As shown in Figure 1, the number of markers per chromosome in the consensus map ranged from 27 to 66 SNPs (with a mean of 43); map length per chromosome ranged from 126 to 207 cM, with a mean of 147 cM. The map distance between markers in the final consensus map ranged from 1.0 to 27.8 cM and the overall mean was 3.5 cM, which is much smaller than the overall mean distance (6.1 cM) of the population specific maps. All except 9 intervals had a map distance < 10 cM (Figure 2).

**Table 3:** Summary of the 18 bi-parental mapping populations used in the present study

**Population code**	**Population type**	**Cross**	**Managed water stressed evaluation sites**	**Well watered evaluation sites**	**Population size**	**No. of SNPs used for genotyping**	**Total number of SNPs mapped**	**Total map length (cM)**
6x1008	F_2:3_	CZL00009/CML505	Chisumaban, Isinya, Kibokooko and Nanga	Embu, Kakamega, Kiboko and Mtwapa	165	201	195	1400
6x1015	F_2:3_	CZL04003/CZL00009	Isinya, Kibokooko and Nanga	Embu, Kakamega, Kiboko and Mtwapa	162	190	179	1227
6x1016	F_2:3_	CZL00009/CZL99017	Isinya, Kibokooko and Nanga	Embu, Kakamega, Kiboko and Mtwapa	148	191	171	1342
6x1017	F_2:3_	CZL00009/CML539	Isinya, Kibokooko and Nanga	Embu, Kakamega, Kiboko and Mtwapa	184	210	199	1305
6x1018	F_2:3_	CML505/CZL99017	Kibokooko and Nanga	Embu, Kakamega, Kiboko and Mtwapa	184	212	177	1333
6x1019	F_2:3_	CZL04008/CZL0719	Kibokooko and Nanga	Embu, Kakamega, Kiboko and Mtwapa	173	202	182	1344
6x1020	F_2:3_	CZL0723/CZL0724	Kibokooko and Nanga	Embu, Kakamega, Kiboko and Mtwapa	181	218	196	1418
6x1021	F_2:3_	CZL0723/CZL0719	Isinya, Kibokooko and Nanga	Embu, Kakamega, Kiboko and Mtwapa	184	217	202	1376
6x1023	F_2:3_	CZL0618/VL062655	Chisumaban, Isinya, Kibokooko and Nanga	Embu, Kakamega, Kiboko and Mtwapa	184	225	200	1351
6x1024	F2:3	CZL02001/VL062590	Chisumanje, Kiboko and Nanga	Embu, Kakamega, Kiboko and Mtwapa	181	204	176	1246
6x1028	F_2:3_	CZL074/VL062645	Chisumabans and Kibokooko	Embu, Kakamega, Kiboko and Mtwapa	174	205	184	1166
6x1115	BC_1_F_3_	CKL09004/CZL00003//CKL09004	Isinya, Chiredzi, Chisumanje, Kiboko and Mtwapa	Kiboko, Kti and Kakamega	184	166	144	1034
6x1116	BC_1_F_3_	CKL09007/CML395//CML395	Isinya, Chiredzi, Chisumanje, Kiboko and Mtwapa	Kiboko, Kti and Kakamega	184	185	145	426
6x1117	BC_1_F_3_	CKL09007/CML444//CML444	Isinya, Chiredzi, Chisumanje, Kiboko and Mtwapa	Kiboko, Kti and Kakamega	160	163	152	459
6x1118	BC_1_F_3_	CKL09001/CML444//CML444	Isinya, Chiredzi, Chisumanje, Kiboko and Mtwapa	Kiboko, Kti and Kakamega	178	177	164	502
6x1120	F_2:3_	CKL09008/CML395	Isinya, Chiredzi, Chisumanje, Kiboko and Mtwapa	Kiboko, Kti and Kakamega	173	166	164	1180
6x1121	BC_1_F_3_	CKL09002/CZL03011//CKL09002	Isinya, Chiredzi, Chisumanje, Kiboko and Mtwapa	Kiboko, Kti and Kakamega	176	173	118	656
6x1122	BC_1_F_3_	CKL09006/CZL03011//CKL09006	Isinya, Chiredzi, Chisumanje, Kiboko and Mtwapa	Kiboko, Kti and Kakamega	155	185	153	581

### QTL in individual populations

**Figure 3:**
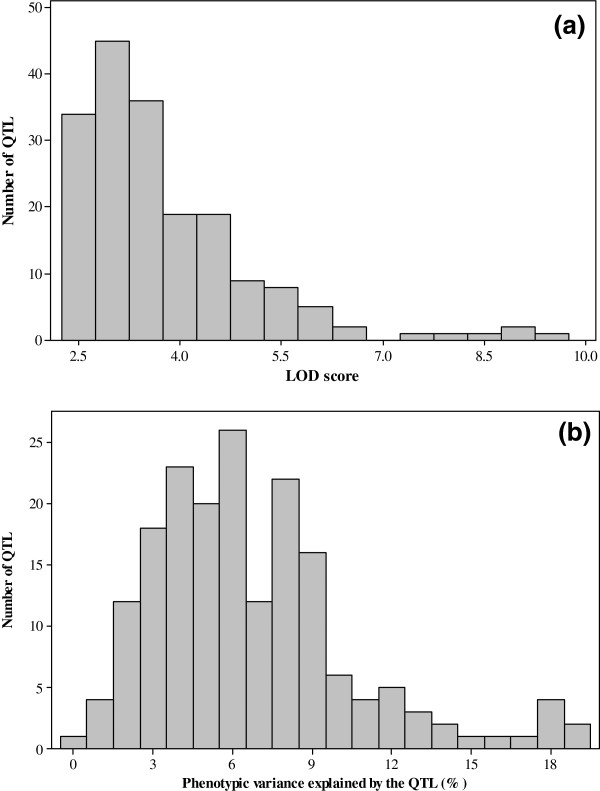
**Frequency distribution of the 183 the 183 GY and ASI QTL by a) LOD score and b) R**^**2**^**.**

**Figure 4:**
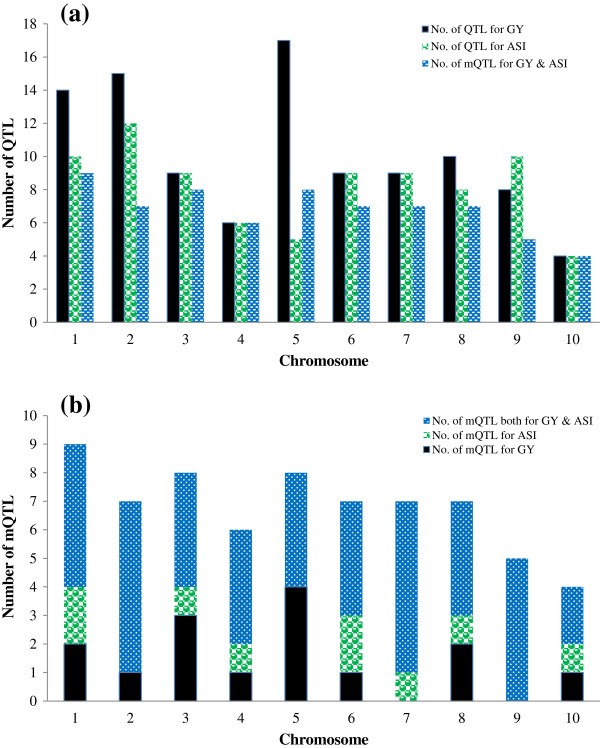
Frequency distribution of (a) population–specific QTL used for projection and total number of mQTL, and (b) the number of mQTL per chromosome.

From the 18 studies, composite interval mapping (CIM) uncovered a total of 101 QTL for GY and 82 QTL for ASI (Tables 1, 2 and Additional file 1). Figures 3 and 4 show the frequency distribution of the QTL for GY and ASI by LOD score and phenotypic variance explained by each QTL and chromosome. Under stressed environments, 18 QTL for GY were uncovered (Table 1) in 6 of the 12 populations with heritability > 0.10 (range: 0-9; average = 1.5 QTL per population). The proportion of phenotypic variance explained by each GY QTL under stress environments varied from 1.3 to 8.4%, and there were between 1 and 4 QTL per chromosome (except chromosome 10). The average phenotypic and genotypic variance explained by each GY QTL under stressed environments was 4.6% and 17.1%, respectively. In the optimum environments, a total of 83 QTL for GY were detected across all 18 populations. The number of GY QTL per population under optimum environments varied from 1 to 7 with an average of 4.6 QTL, and each QTL explained 1.2 to 19.1% of the phenotypic variance. The QTL were distributed across all chromosomes with the number of QTL per chromosome ranging from 4 to 14, with a mean of 4.8 QTL. The mean phenotypic and genotypic variance explained by each GY QTL under optimum environments was 7.7% and 18.1%, respectively.

For ASI, a total of 33 QTL with heritability > 0.10 were uncovered in 11 populations under stressed environments (Table 2 and Additional file 1). Each QTL for ASI explained 0.1 to 12.1% of the phenotypic variance under water stress, and they were distributed across all chromosomes with each chromosome containing from 1 to 6 QTL, (an average of 3.3 QTL per population). The mean phenotypic and genotypic variance explained by each QTL for ASI under stress was 5.0% and 22.7%, respectively. QTL for ASI with heritability > 0.20 were detected in 13 of the 16 populations under optimum environments, with the number of QTL varying from 1 to 7 and an average of 3.8 QTL per population. Each QTL for ASI explained 1.1 to 17.3% of the phenotypic variance under optimum environments. The QTL for ASI under optimum environments were distributed across all chromosomes with the number of QTL per chromosome ranging from 2 to 8 and an average of 5.1 per chromosome. The average phenotypic and genotypic variance explained by each QTL for ASI under optimum environments was 6.7% and 18.7%, respectively. Several QTL for GY and ASI had overlapping confidence intervals, and they appeared in clusters in the linkage maps (Additional files 1 and 2).

### Meta-analyses

**Figure 5:**
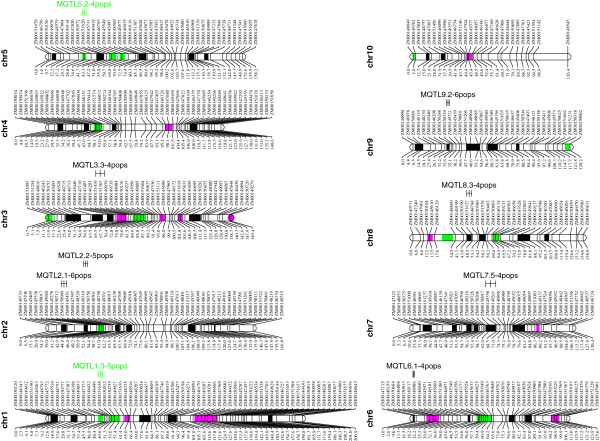
**The positions of the 68 mQTL for grain yield and anthesis silking interval.** The 95% genetic confidence interval of mQTL for grain yield and anthesis silking interval are shaded in green and pink colors, respectively, while those shaded in black coincided for both traits. The mQTL detected in >4 populations are indicated on the right side of each chromosome.

All QTL identified in individual populations were projected on the consensus map separately for GY and ASI first, and then for the combined QTL results of both traits (Additional file 2). The analysis of the combined traits increased the number of QTL per chromosome from a range of 4-17 to a range of 8-27. The statistical power via single trait-analysis and combined traits analyses was the same (data not shown). The meta-analysis sharply reduced the total number of QTL from 183 to 68 mQTL, compared to individual populations (Figure 4). Nine of these mQTL were specific to ASI, 15 to GY, and the remaining 44 were common to both GY and ASI (Figures 4 and 5). Table 4 and Additional file 3 present information about each mQTL, including chromosomal position, genetic and physical confidence interval, R^2^, flanking markers, and number of candidate genes in the interval. Eight of the 68 mQTL were associated either with ASI (5 mQTL) or both ASI and GY (3 mQTL) under water stressed environments only. The other 28 mQTL were detected both under stressed and optimum environments and the remaining 32 mQTL were associated with GY, ASI or both traits under optimum environments only.

**Table 4:** Summary of the 68 meta QTL (mQTL) for grain yield and anthesis-silking interval detected across 18 maize populations

**Chromosome**	**Meta QTL name**	**K**	**Predicted meta QTL position (cM)**	**Meta QTL 95% genetic confidence interval (cM)**	**Initial number of QTL**	**Mean initial confidence interval (cM)**	**Mean LOD score of the initial QTL**	**Mean R**^**2 **^**for the initial QTL**	**Δ in the 95% confidence interval (cM)**	**95% physical confidence interval (kb)**	**Physical distance (kb)**	**No. of candidate genes within the physical interval**	**No. of populations where the meta QTL was uncovered**	
1	MQTL1.1	1	4.3	5.3	3	9.3	3.5	7.7	4.0	3639-4732	1,093.0	59	3	
1	MQTL1.2	2	20.5	5.0	3	12.7	2.9	4.3	7.7	10061-14464	4,402.1	213	3	
**1**	**MQTL1.3**	**3**	**42.2**	**3.8**	**5**	**14.4**	**3.3**	**9.3**	**10.6**	28552-30583	**2,031.9**	**72**	**5**	
1	MQTL1.4	4	53.9	7.5	3	13.3	3.5	7.8	5.8	45277-46989	1,711.8	73	3	
1	MQTL1.5	5	64.0	4.0	1	4.0	4.3	5.9	0.0	51515-58369	6,854.6	227	1	
1	MQTL1.6	6	78.5	9.1	2	16.0	3.2	6.8	7.0	70862-79893	9,031.5	257	2	
1	MQTL1.7	7	100.8	7.1	4	16.0	3.5	7.1	8.9	191405-198269	6,864.1	227	3	
1	MQTL1.8	8	128.0	9.0	1	18.0	2.5	12.3	9.0	223836-229962	6,125.7	234	1	
1	MQTL1.9	9	180.8	3.3	2	12.0	2.9	4.0	8.7	284698-288173	3,475.7	145	1	
**2**	**MQTL2.1**	**1**	**12.4**	**4.5**	**6**	**14.0**	**3.9**	**8.0**	**9.5**	3534-3847	**313.2**	**25**	**6**	
**2**	**MQTL2.2**	**2**	**30.2**	**3.3**	**5**	**10.8**	**3.1**	**5.8**	**7.6**	6141-6957	**815.7**	**48**	**5**	
2	MQTL2.3	3	42.2	4.7	2	6.0	5.8	9.6	1.3	9969-11272	1,302.3	71	2	
2	MQTL2.4	4	56.2	3.5	3	8.7	3.8	6.6	5.2	18063-19837	1,774.0	68	3	
2	MQTL2.5	5	65.7	4.4	4	11.0	4.1	7.1	6.6	28812-37684	8,871.8	322	3	
2	MQTL2.6	6	116.7	5.1	3	13.3	3.4	4.4	8.3	209512-214904	5,391.9	268	3	
2	MQTL2.7	7	135.4	0.6	4	8.0	3.5	2.8	7.4	226386-230734	4,347.5	287	2	
3	MQTL3.1	1	2.0	3.6	2	6.0	4.0	4.5	2.4	1384-1699	315.4	32	2	
3	MQTL3.2	2	20.7	12.5	2	21.0	4.4	6.4	8.5	3266-5558	2,291.9	108	2	
**3**	**MQTL3.3**	**3**	**42.7**	**7.3**	**5**	**20.8**	**3.5**	**5.8**	**13.5**	7218-10471	**3,253.7**	**138**	**4**	
3	MQTL3.4	4	54.5	4.7	2	7.0	3.3	7.2	2.3	27986-53834	25,847.8	619	2	
3	MQTL3.5	5	74.6	10.5	2	15.0	3.5	7.3	4.5	141780-165485	23,704.8	660	2	
3	MQTL3.6	6	93.5	4.6	3	10.7	3.8	6.5	6.1	175055-197483	22,428.3	926	3	
3	MQTL3.7	7	108.0	4.0	1	4.0	3.6	6.7	0.0	208947-211212	2,264.7	92	1	
3	MQTL3.8	8	120.0	9.4	1	12.0	3.9	3.6	2.6	211133-218712	7,578.4	308	1	
4	MQTL4.1	1	9.5	4.5	3	8.7	3.1	4.2	4.2	1613-4989	3,376.2	206	3	
4	MQTL4.2	2	30.8	4.1	3	7.3	3.2	6.1	3.2	11272-13790	2,518.2	87	3	
4	MQTL4.3	3	40.0	6.0	1	6.0	3.6	1.2	0.0	14511-18502	3,991.4	125	1	
4	MQTL4.4	4	53.4	3.3	2	5.0	6.2	12.7	1.7	41714-81616	39,902.1	851	1	
4	MQTL4.5	5	98.0	6.0	1	6.0	4.0	6.5	0.0	196386-203074	6,688.0	238	1	
4	MQTL4.6	6	118.2	6.0	2	14.0	3.0	4.4	8.0	240863-242930	2,067.0	18	2	
5	MQTL5.1	1	4.2	3.3	3	13.3	2.7	3.4	10.0	887-2799	1,911.3	189	2	
**5**	**MQTL5.2**	**2**	**28.9**	**3.1**	**4**	**8.0**	**3.9**	**8.5**	**5.0**	6821-9201	**2,379.9**	**128**	**4**	
5	MQTL5.3	3	42.0	6.0	1	6.0	3.9	5.3	0.0	11666-13316	1,650.1	83	1	
5	MQTL5.4	4	52.0	5.7	2	8.0	2.6	5.9	2.3	21458-42985	21,527.0	645	2	
5	MQTL5.5	5	60.0	4.0	1	4.0	4.9	9.9	0.0	46400-75946	29,545.5	863	1	
5	MQTL5.6	6	76.4	6.3	3	11.3	3.7	7.7	5.0	158664-169720	11,056.0	324	3	
5	MQTL5.7	7	116.4	4.7	3	9.3	3.4	5.0	4.6	199917-203728	3,810.3	191	3	
5	MQTL5.8	8	128.6	3.2	5	20.5	4.3	5.5	17.3	204095-208963	4,867.9	305	3	
**6**	**MQTL6.1**	**1**	**0.4**	**1.9**	**4**	**8.0**	**3.1**	**4.0**	**6.1**	1535-3654	**2,119.7**	**66**	**4**	
6	MQTL6.2	2	16.0	10.0	1	10.0	3.7	6.0	0.0	7800-21710	13,909.7	301	1	
6	MQTL6.3	3	45.8	1.9	3	8.0	4.2	10.5	6.1	109410-109818	407.2	5	3	
6	MQTL6.4	4	58.0	12.0	1	12.0	2.7	5.2	0.0	115280-138426	23,145.6	829	1	
6	MQTL6.5	5	80.6	4.7	4	12.5	4.6	8.8	7.8	153123-156645	3,521.2	201	3	
6	MQTL6.6	6	94.2	5.4	4	11.0	4.6	9.6	5.6	160735-161977	1,241.9	77	2	
6	MQTL6.7	7	116.0	6.5	1	10.0	2.9	1.5	3.5	163973-165726	1,753.2	116	1	
7	MQTL7.1	1	11.6	7.2	2	12.0	6.4	11.5	4.8	1977-3000	1,023.8	71	2	
7	MQTL7.2	2	33.9	3.7	4	15.0	3.2	5.3	11.3	7344-8456	1,112.2	51	3	
7	MQTL7.3	3	45.6	4.0	3	8.0	4.1	8.7	4.0	11348-17346	5,998.1	177	2	
7	MQTL7.4	4	51.5	3.3	2	5.0	3.1	5.8	1.7	92209-111874	19,664.7	387	2	
**7**	**MQTL7.5**	**5**	**63.4**	**8.2**	**4**	**16.5**	**3.4**	**6.8**	**8.3**	119475-128209	**8,734.7**	**289**	**4**	
7	MQTL7.6	6	86.4	9.6	2	14.0	3.4	4.4	4.4	155968-156660	692.0	28	1	
7	MQTL7.7	7	102.0	2.3	1	6.0	3.6	4.2	3.7	162969-163528	559.5	23	1	
8	MQTL8.1	1	14.0	4.0	1	4.0	2.6	2.2	0.0	8255-12884	4,628.6	174	1	
8	MQTL8.2	2	28.2	8.7	2	14.0	7.0	12.9	5.3	12884-16036	3,152.4	118	2	
**8**	**MQTL8.3**	**3**	**45.8**	**4.0**	**5**	**11.6**	**4.0**	**7.8**	**7.7**	19226-22594	**3,368.0**	**112**	**4**	
8	MQTL8.4	4	56.6	4.9	3	11.3	3.4	7.3	6.4	94597-114908	20,310.3	572	3	
8	MQTL8.5	5	68.0	4.2	2	6.0	4.1	11.2	1.8	130389-136848	6,459.5	238	2	
8	MQTL8.6	6	90.5	10.1	2	15.0	4.2	7.9	4.9	161536-162181	645.2	39	2	
8	MQTL8.7	7	109.2	3.7	3	14.7	3.2	4.7	11.0	165872-166992	1,119.5	67	3	
9	MQTL9.1	1	4.9	6.7	3	12.0	3.0	4.1	5.4	558-1039	480.9	17	2	
**9**	**MQTL9.2**	**2**	**28.6**	**2.5**	**6**	**10.7**	**3.4**	**3.5**	**8.3**	12863-15939	**3,076.3**	**107**	**6**	
9	MQTL9.3	3	48.9	10.7	2	16.0	3.3	4.7	5.3	78943-107127	28,183.8	676	2	
9	MQTL9.4	4	64.9	8.3	3	14.7	4.2	7.1	6.4	122950-134502	11,552.0	424	3	
9	MQTL9.5	5	85.6	2.5	4	13.0	3.8	5.2	10.5	139422-142013	2,590.7	118	3	
10	MQTL10.1	1	0.0	4.0	1	4.0	2.9	1.5	0.0	5116-5692	576.2	27	1	
10	MQTL10.2	2	12.0	2.7	3	5.3	4.3	9.4	2.6	7133-13608	6,475.7	192	2	
10	MQTL10.3	3	24.7	3.5	3	6.0	7.0	13.1	2.5	15276-62174	46,897.5	857	3	
10	MQTL10.4	4	46.0	4.2	1	8.0	4.0	6.5	3.8	127259-131505	4,245.7	160	1	

See Additional file 3 for more information.

The number of mQTL identified on each chromosome varied from 4 on chromosome 10 to 9 on chromosome 1, with an average of 6.8 mQTL per chromosome. The low marker density on chromosome 10 may have reduced the number of mQTL uncovered on this chromosome. The mean phenotypic variance explained by each mQTL varied from 1.2 to 13.1% and the overall average was 6.5%. The 95% genetic confidence intervals for the mQTL varied between 0.6 and 12.5 cM, with an average of 5.4 cM, which is half the sizes of their respective original QTL (range = 4.0-21.0 cM; average = 10.7 cM). The 95% physical confidence intervals ranged from 313 to 46,898 kb with an average of 7,574 kb. The total number of candidate genes within the physical intervals varied from 5 to 926, with an average of 239 candidate genes per mQTL (Table 4). The physical to genetic distance ratio varied from 64 to 13,554 kb/cM, and the average was 7,574 kb/cM.

Eighteen of the 68 mQTL were detected in only a single population, 20 mQTL in 2 populations, 21 mQTL in 3 populations, 5 mQTL in 4 populations, 2 mQTL in 5 populations, and 2 mQTL in 6 populations (Table 4). No mQTL was detected in more than 6 of the 18 populations. Among the 9 mQTL mapped in 4-6 populations, four mQTL (mQTL2.2, mQTL6.1, mQTL7.5 and mQTL9.2) were associated with GY under both stress and optimum conditions; 2 mQTL (mQTL1.3 and mQTL5.2) were associated with GY only under optimum conditions; and the remaining 3 mQTL (mQTL2.1, mQTL3.3 and mQTL8.3) were associated with GY under optimum and ASI under water stressed and/or optimum environments. MQTL2.2 is located on chromosome 2 between 28.6 and 31.8 cM and has a physical interval of 816 kb. This QTL explains on average 5.8% of the phenotypic variance for GY both under water stress and optimum environments and encompassed 48 candidate genes. MQTL6.1 is located at the proximal end of chromosome 6 and has a physical interval of 2,120 kb; it accounted on average for 4.0% of the phenotypic variance for GY both under water stress and optimum environments and encompassed 66 candidate genes. MQTL7.5 is located on chromosome 7 between 59.4 and 67.5 cM and has a physical interval of 1,069 kb; this QTL explains on average 6.8% of the phenotypic variance for GY both under water stress and optimum environments and encompassed 289 candidate genes. MQTL9.2 is located on chromosome 9 between 27.4 and 29.8 cM and has a physical interval of 12,569 kb. The latter mQTL explains on average 3.5% of the phenotypic variance for GY both under water stress and optimum environments and encompassed 107 candidate genes.

## Discussion

The projection of many QTL on a consensus map for meta-analysis allows to ascertain whether the QTL detected under water stressed conditions are a subset of those detected under optimal conditions, and if the QTL are common across different mapping populations (genetic backgrounds). Our study clearly demonstrated four times as many mQTL expressed under optimum conditions than under stressed environments. There was lower broad-sense heritability both for GY and ASI under stressed (0.13 to 0.40) than under optimum (0.21 to 0.58) environments, which may be an indication of a larger environmental component to the variance associated with stressed compared to optimum conditions. Although heritability under stress is sometimes comparable with heritability under optimum conditions, many studies [25-27] have also reported lower heritability under water stress than under optimum conditions. The mQTL were very specific to genetic background, but 8 of the 9 mQTL (MQTL1.3, MQTL2.1, MQTL2.2, MQTL3.3, MQTL5.2, MQTL6.1, MQTL8.3 and MQTL9.2; Table 4) found in 4 to 6 populations had small to medium genetic (1.9-8.2 cM) and physical (313-3368 kb) intervals and may be important regions for marker assisted backcrossing, QTL cloning for transformation, and/or functional analysis. Four of these mQTL (mQTL2.2, mQTL6.1, mQTL7.5 and mQTL9.2) seem the most suitable for future studies and eventual incorporation into breeding lines because (a) they were associated with GY under both water stressed and optimum environments; (b) they were detected up to 6 genetic backgrounds; (c) they accounted on average 3.5 to 6.8% of the phenotypic variance for GY under stress and optimum conditions, and (d) they encompassed lower number (48 to 289) of candidate genes.

Candidate genes can be identified through positional cloning using QTL confidence intervals [10], but confidence intervals need to be as small as possible. Combining results from several genome-wide surveys [28] and/or by merging QTL data from different studies [29] can help accomplish this. Our results from meta-analysis clearly demonstrated a gain in precision, reaching up to 12-fold smaller confidence interval in the mQTL, as compared with the population specific maps (Table 4). Similar results have been reported in other studies [20,23,24,30,31]. As shown in Table 4, the number of candidate genes within the 4 most conserved mQTL associated with GY both under water stressed and optimum environments varied from 48 to 289, and will thus require a further shrinking by fine mapping. This can most easily be done by increasing marker density evenly in the target regions, for example via genotyping-by-sequencing (GBS), which will generate nearly a million SNPs per sample at a cost of about $22 to $38 (http://igd.cornell.edu/index.cfm/page/GBS/GBSpricing.htm). At least 10% of these SNPs are expected to be polymorphic between parents in a given cross, and will thus generate at least 100 thousand polymorphic SNPs for fine mapping. The mapping populations presented in this study have been submitted for GBS, which may narrow down the physical confidence interval of the mQTL. Further fine mapping and/or QTL validation can also be done by increasing the size of the mapping populations. The four mQTL for GY detected in multiple populations under both stressed and optimum environments were also associated with ASI under stress and/or optimum conditions. However, we are unsure if this was due to the pleiotropic action of a single gene or multiple linked genes [16,32]. If the cause is tight linkage of multiple genes, fine mapping of large numbers of recombinants will break up the linkage. Although this is a labor and time-consuming process, it will be proposed for the conserved mQTL of large phenotypic effect.

Some of the mQTL detected in this study explained up to 13% of the phenotypic variance for GY and ASI under stress and/or optimum conditions. Because each mapping populations had an average of 174 progenies, it is possible that some of the mQTL of large effect may showed upward biased estimation (Beavis effect) of the phenotypic effects [33,34]. MQTL with large physical intervals may also contain several linked genes influencing the same trait. This has been reported even in cases where QTL effects have been fine mapped to more than one specific gene [32]. As far as we are aware, this is the first study that reports extensive mQTL results using over 3100 individuals that were genotyped using the same SNP platform and phenotyped in the same way across a wide range of managed water stressed and well watered environments. Future investigations may involve fine mapping and/or verification of some of the mQTL regions detected across 4-6 populations using large population size and high marker density. The results from this study provide highly valuable information for researchers working on QTL mapping for possible use in marker assisted selection and/or QTL cloning.

## Conclusions

Meta-analyses reduced the number of QTL by 68% and narrowed the confidence intervals up to 12-fold, but none of the mQTL were detected in more than 6 populations, confirming the uniqueness of QTL from different populations. Nevertheless, at least 4 of the 68 mQTL were detected at least in 4 populations and may be considered for fine mapping and validation using large population sizes and high marker density, such as GBS. These four mQTL were located on chromosomes 2 (mQTL2.2), chromosome 6 (mQTL6.1), chromosome 7 (mQTL7.5) and chromosome 9 (mQTL9.2). About 65% of the mQTL uncovered under water stressed and/or optimum environments coincided between grain yield and ASI but it is unclear whether such large number of coincident mQTL was due to pleiotropic effect or tight linkage.

## Methods

### Population development, phenotyping and genotyping

A total of 25 MARS populations were initiated in 2008 and 2009. Quality control (QC) genotyping [35] of F_1_s and their parents with 100 SNP markers identified all F_1_s with true-to-type parental alleles for ≥ 95% of the polymorphic SNPs for advancement either to F_2:3_ or BC_1_F_3_, while those with >5% non-parental alleles were discarded. Seven of the 25 MARS populations either failed to pass the quality control genotyping criteria or had broad sense heritability < 0.10 and/or < 0.20 in the combined analyses of all the stressed and optimum environments, respectively, and were excluded from analyses. Phenotypic evaluations were performed on testcrosses derived by crossing either the F_2:3_ or BC_1_F_3_ families with one single cross tester from opposite heterotic group. The parents crossed with the same tester, and selected commercial checks were included in each of the trials. Each population was planted using an alpha lattice design, with 2 replications per location, and evaluated in 2-4 managed water stressed and 3-4 well watered locations (Table 3). Each entry was planted in a 5 m long row with spacing of 0.75 m between rows and 0.25 m between plants. In maize, it is well known that grain yield is often reduced 2-3 times more when water deficits coincide with flowering, compared with other growth stages [36]. Therefore, water stress evaluation was conducted during the dry (rain free) season in Kenya, Zimbabwe and Zambia by withdrawing irrigation two weeks before flowering. Irrigation was resumed at the end of the flowering stage, corresponding to the end of silk emergence, and maintained until harvest to allow grain filling. Evaluation under optimum conditions in the 3 countries was carried out during the long rainy season.

Each population was evaluated for 12-17 different traits, including grain yield, anthesis date, number of ears per plant, and leaf senescence, which are commonly associated with drought tolerance. Only grain yield and ASI were selected as the main target traits in the present study. ASI was computed as the difference between days to silking and anthesis. Each trial was harvested when all leaves had senesced. Ears were dried and shelled, grain was weighed, and grain moisture determined by a capacitance meter. SAS program v9.2 was used for phenotypic data analyses, including calculating Best Linear Unbiased Predictor (BLUP), variance components and heritability under stressed and optimum environments.

### Linkage and QTL mapping in individual populations

All mapping populations were genotyped by the Monsanto Company using a TaqMan assay (http://www.appliedbiosystems.com). For each segregating SNP, a χ2 goodness-of-fit analysis was performed to test for deviation from the expected segregation ratio. The chromosomal position and locus order of all SNPs used in the present study was provided by the Monsanto Company and this *a priori* information was used as a reference for determining locus order in our mapping populations. Linkage groups were established using LOD scores ranging between 3 and 15, and recombination frequency of 0.30. The order of the SNPs on each chromosome was determined as described elsewhere [37] using the Kosambi mapping function. χ2 analyses and linkage mapping were performed using JoinMap version 4.0 [38]. The number of polymorphic SNPs used for genotyping the populations varied from 163 to 225 (Table 3). Final linkage maps were constructed after excluding a total of 389 non-informative SNPs (an average of 22 SNPs per population) because they i) did not meet the threshold value for goodness-of-fit, ii) contributed to negative distance in the final map, iii) changed the expected marker order, or iv) mapped to unexpected chromosomal locations compared to the *a priori* information. QTL mapping was performed with BLUP values obtained across the combined analyses of all the stressed and optimum environments for each population. Composite interval mapping (CIM) was conducted as described elsewhere [39] using a minimum LOD score of 2.5 and the PLABQTL software, version 1.2 [40,41].

### Map projection and QTL meta-analyses

For the same chromosome across multiple populations, a consensus linkage map of all SNPs was constructed from the population specific maps using BioMercator version 2.1 as described by Arcade et al. [42]. Markers that showed inversions in the consensus map were discarded. The initial consensus map consisted of 961 markers but about 55% of the SNPs (531 of the 961 SNPs) had a map distance < 1 cM to adjacent markers, so they were excluded from the final consensus map. All QTL identified in individual populations using PlabQTL were projected on the consensus map separately for GY and ASI first, and then for the combined QTL results of both traits. The information on the original chromosomal position, LOD score, confidence interval (CI) and proportion of phenotypic variance (R^2^) explained by each QTL (as summarized in Additional file 1) were used for the projection. For each chromosome, meta-analysis was used to estimate the numbers, positions, and 95% confidence interval of the mQTL using BioMercator version 3.0 software (http://moulon.inra.fr/index.php/en/scientific-output/software/doc_details/15-biomercator-v-3) [43]. The meta-analysis first determines the best model based on model choice criteria of the following: AIC (Akaike information criterion), AICc, AIC3, BIC (Bayesian information criterion) and AWE (average weight of evidence). The best QTL model was selected when values of the model selectin criteria were the lowest at least in 3 of the 5 models (Additional file 4). The best model was then used in the MQTLView method. QTL with probability of membership in a given mQTL > 60% were assigned to the same mQTL. The 95% confidence intervals of the mQTL were drawn using the MapChart program, version 2.1 [44].

### Candidate genes

Flanking markers of each mQTL were used to search for candidate genes within each mQTL interval. The genetic map of all proprietary SNPs used in this study, along with over 52,000 public markers, was provided by the Monsanto Company. The map was created using the company’s proprietary mapping population. For each mQTL, the public markers with known physical positions that were closest to the two flanking SNPs found in this study were chosen to define the interval. The physical positions of these flanking public markers were then used to search for candidate genes using the Maize Sequence database (http://www.maizesequence.org/index.html). This browser provides the latest sequence and annotation of the *Zea mays* ssp. *mays* genome from the Maize Genome Sequencing Project.

## Competing interest

The authors declare no competing financial interests.

## Authors’ contributions

KS was responsible for data analyses and writing the manuscript; YB was responsible in population development, experimental design and overall multi-location phenotyping of the populations; AT coordinated phenotyping of the populations both in Zambia and Zimbabwe; SM contributed in the project planning and overall coordination; BM and PS were responsible for coordinating sample management, polymorphic markers selection, DNA extraction and genotyping, and IT requirements at the Monsanto company; both MLW and BMP contributed to the data analyses and edited the manuscript. All authors have made their contribution in editing the manuscript and approved the final version.

## Supplementary Material

Additional file 1 Summary of the population-specific QTL detected by Composite Interval Mapping for grain yield (GY) and anthesis-silking interval (ASI) for 18 maize populations evaluated under managed water stressed (WS) and well-watered (WW) environments. Click here for file

Additional file 2 Summary of the projected position of the 183 QTL for grain yield (GY) and anthesis-silking interval (ASI) using BioMercator version 3.0. Click here for file

Additional file 3 Additional information to the meta QTL described in Table 4. Click here for file

Additional file 4 Summarizes the model selection criteria in the meta-analyses. Click here for file
