# Supplementary material for: Meta-analyses of QTL for grain yield and anthesis silking interval in 18 maize populations evaluated under water-stressed and well-watered environments
Source: BMC Genomics. 2013 May 10;14:313. doi: 10.1186/1471-2164-14-313 (PMC3751468; doi:10.1186/1471-2164-14-313)
Supplement: Additional file 1 — Summary of the population-specific QTL detected by Composite Interval Mapping for grain yield (GY) and anthesis-silking interval (ASI) for 18 maize populations evaluated under managed water stressed (WS) and well-watered (WW) environments. [file 1471-2164-14-313-S1.pdf]

Additional data file 1: Summary of the population-specific QTLs found by Composite Interval Mapping for grain yield (GY) and anthesis-silking interval (ASI) for 18 maize populations evaluated under managed water stressed (WS) and well-watered (WW) environments. See Table 3 for details in population code.

S/N	Population code	Original QTL name	Chromosome	Trait	Environment	LOD score	R^2	QTL position (cM)	Confidence interval (cM)	Left flanking marker	Right flanking marker	Parental source
1	6x1028	GY1	1	GY	WW	4.3	9.0	2.0	0_8	ZM00150120	ZM00150927	CZL074
2	6x1116	ASI1	1	ASI	WW	2.9	8.4	4.0	0_10	ZM00150120	ZM00148041	CKL09007
3	6x1008	ASI2	1	ASI	WW	3.4	5.6	8.0	4_14	ZM00149412	ZM00149263	CML505
4	6x1122	ASI3-WS	1	ASI	WS	2.9	5.6	18.0	16_22	ZM00150142	ZM00146451	CZL03011
5	6x1020	GY2	1	GY	WW	2.8	2.6	26.0	20_30	ZM00151177	ZM00147847	CZL0723
6	6x1024	GY3	1	GY	WW	2.9	4.6	26.0	12_34	ZM00150777	ZM00151649	CZL02001
7	6x1018	GY4	1	GY	WW	3.6	6.3	32.0	16_48	ZM00145772	ZM00147873	CZL99017
8	6x1021	GY5	1	GY	WW	3.1	5.8	38.0	34_44	ZM00149853	ZM00148432	CZL0719
9	6x1117	GY6	1	GY	WW	3.8	5.0	42.0	38_44	ZM00145997	ZM00151635	CML444
10	6x1016	GY7	1	GY	WW	3.1	10.3	44.0	36_54	ZM00151046	ZM00150077	CZL99017
11	6x1121	GY8	1	GY	WW	3.2	18.9	44.0	40_46	ZM00145477	ZM00150142	CZL03011
12	6x1015	GY9	1	GY	WW	3.9	6.9	54.0	44_56	ZM00147873	ZM00147847	CZL04003
13	6x1017	GY10	1	GY	WW	3.5	9.3	54.0	46_58	ZM00151649	ZM00148432	CZL00009
14	6x1020	GY11	1	GY	WW	3.2	7.1	54.0	44_60	ZM00148079	ZM00151207	CZL0723
15	6x1122	ASI4	1	ASI	WW	4.3	5.9	64.0	62_66	ZM00148343	ZM00151635	CZL03011
16	6x1023	ASI5	1	ASI	WW	3.1	5.9	78.0	72_82	ZM00150654	ZM00150417	CZL0618
17	6x1021	GY12	1	GY	WW	3.3	7.6	80.0	66_88	ZM00148335	ZM00150417	CZL0723
18	6x1019	GY13	1	GY	WW	3.2	4.7	92.0	84_104	ZM00148267	ZM00150417	CZL04008
19	6x1028	ASI6	1	ASI	WW	3.4	9.0	98.0	86_106	ZM00150636	ZM00151640	VL062645
20	6x1020	ASI7	1	ASI	WW	2.9	5.9	100.0	92_106	ZM00147369	ZM00150566	CZL0723
21	6x1028	ASI8-WS	1	ASI	WS	4.4	8.7	104.0	100_110	ZM00151640	ZM00147369	VL062645
22	6x1020	ASI9	1	ASI	WW	2.5	12.3	128.0	118_136	ZM00145787	ZM00148508	CZL0724
23	6x1021	ASI10	1	ASI	WW	2.7	5.3	160.0	152_168	ZM00151633	ZM00147772	CZL0723
24	6x1021	GY14-WS	1	GY	WS	3.0	2.7	186.0	184_192	ZM00150677	ZM00149480	CZL0723
25	6x1021	GY15	2	GY	WW	3.7	9.1	2.0	0_12	ZM00148261	ZM00148541	CZL0719
26	6x1116	GY16	2	GY	WW	5.1	12.7	8.0	4_12	ZM00150949	ZM00150756	CML395
27	6x1116	ASI11	2	ASI	WW	3.1	3.2	14.0	8_22	ZM00150949	ZM00150756	CKL09007
28	6x1015	GY17	2	GY	WW	4.4	5.1	18.0	6_22	ZM00145538	ZM00149920	CZL00009
29	6x1019	ASI12	2	ASI	WW	3.9	6.9	18.0	6_32	ZM00151215	ZM00145278	CZL0719
30	6x1122	ASI13	2	ASI	WW	3.3	11.0	18.0	12_20	ZM00149920	ZM00145278	CKL09006
31	6x1020	GY18	2	GY	WW	2.6	8.5	22.0	20_36	ZM00150756	ZM00146728	CZL0724
32	6x1028	ASI14	2	ASI	WW	4.3	8.8	24.0	14_30	ZM00150362	ZM00147397	VL062645
33	6x1116	GY19-WS	2	GY	WS	2.7	3.1	26.0	22_30	ZM00145918	ZM00145647	CML395
34	6x1008	GY20-WS	2	GY	WS	3.0	1.3	30.0	26_36	ZM00145278	ZM00150949	CML505
35	6x1122	ASI15	2	ASI	WW	3.0	7.1	32.0	30_34	ZM00145576	ZM00149238	CZL03011
36	6x1018	GY21	2	GY	WW	9.0	11.7	42.0	40_46	ZM00149310	ZM00150756	CZL99017
37	6x1024	GY22	2	GY	WW	2.7	7.5	42.0	36_44	ZM00147397	ZM00148345	VL062590
38	6x1015	GY23	2	GY	WW	5.0	6.1	50.0	44_58	ZM00150756	ZM00148542	CZL00009
39	6x1122	ASI16-WS	2	ASI	WS	3.5	3.6	56.0	54_58	ZM00149124	ZM00148001	CZL03011
40	6x1017	GY24	2	GY	WW	3.0	10.2	58.0	56_64	ZM00148589	ZM00149238	CZL00009
41	6x1023	ASI17	2	ASI	WW	4.9	9.5	62.0	58_66	ZM00150880	ZM00145647	CZL0618
42	6x1023	ASI18-WS	2	ASI	WS	4.1	6.3	66.0	58_70	ZM00150880	ZM00145647	CZL0618
43	6x1028	ASI19-WS	2	ASI	WS	3.1	2.3	66.0	64_70	ZM00149406	ZM00149124	VL062645
44	6x1017	GY25	2	GY	WW	4.4	10.4	80.0	72_90	ZM00145918	ZM00150343	CML539
45	6x1021	GY26-WS	2	GY	WS	5.0	4.5	116.0	114_120	ZM00147812	ZM00147913	CZL0719
46	6x1017	GY27	2	GY	WW	2.6	5.5	118.0	112_122	ZM00148212	ZM00146060	CML539
47	6x1028	ASI20-WS	2	ASI	WS	2.6	3.1	122.0	112_136	ZM00150694	ZM00149315	CZL074
48	6x1008	ASI21-WS	2	ASI	WS	2.8	0.1	128.0	122_132	ZM00145473	ZM00145689	CZL00009
49	6x1008	GY28	2	GY	WW	4.7	5.7	134.0	130_136	ZM00146101	ZM00146228	CML505

50	6x1021	ASI22	2	ASI	WW	2.6	1.1	136.0	134_138	ZM00147991	ZM00151261	CZL0719
51	6x1019	GY29	2	GY	WW	4.0	4.1	146.0	140_152	ZM00147913	ZM00146060	CZL0719
52	6x1019	GY30	3	GY	WW	2.6	4.5	2.0	0_8	ZM00147604	ZM00148816	CZL04008
53	6x1116	GY31	3	GY	WW	5.5	4.4	2.0	0_4	ZM00148816	ZM00148974	CKL09007
54	6x1021	GY32-WS	3	GY	WS	5.9	8.4	20.0	16_30	ZM00147828	ZM00146066	CZL0719
55	6x1008	ASI23-WS	3	ASI	WS	2.9	4.3	22.0	8_36	ZM00148816	ZM00146066	CML505
56	6x1115	GY33	3	GY	WW	2.7	4.8	38.0	18_46	ZM00148713	ZM00146998	CKL09004
57	6x1021	ASI24-WS	3	ASI	WS	2.9	3.9	38.0	24_58	ZM00146066	ZM00145446	CZL0719
58	6x1122	ASI25-WS	3	ASI	WS	2.6	3.9	40.0	34_44	ZM00148067	ZM00146826	CKL09006
59	6x1021	GY34	3	GY	WW	5.2	13.0	48.0	38_58	ZM00145446	ZM00147996	CZL0723
60	6x1028	ASI26-WS	3	ASI	WS	4.0	3.2	48.0	38_50	ZM00148816	ZM00145446	VL062645
61	6x1023	GY35	3	GY	WW	3.5	7.8	52.0	48_54	ZM00149582	ZM00147407	VL062655
62	6x1021	ASI27	3	ASI	WW	3.0	6.6	60.0	58_66	ZM00147996	ZM00145332	CZL0719
63	6x1019	GY36	3	GY	WW	2.9	5.8	72.0	66_80	ZM00149385	ZM00147285	CZL04008
64	6x1120	GY37	3	GY	WW	4.1	8.7	78.0	70_86	ZM00148770	ZM00148805	CML444
65	6x1008	ASI28	3	ASI	WW	5.1	8.8	92.0	90_96	ZM00150837	ZM00148897	CML505
66	6x1024	ASI29	3	ASI	WW	2.7	4.9	94.0	92_110	ZM00148897	ZM00145344	CZL02001
67	6x1028	ASI30	3	ASI	WW	3.5	5.9	96.0	92_100	ZM00145699	ZM00147965	CZL074
68	6x1020	ASI31	3	ASI	WW	3.6	6.7	108.0	106_110	ZM00147092	ZM00147809	CZL0724
69	6x1115	GY38	3	GY	WW	3.9	3.6	120.0	114_126	ZM00147590	ZM00149093	CZL00003
70	6x1116	GY39-WS	4	GY	WS	2.8	3.8	8.0	6_12	ZM00150249	ZM00147956	CML395
71	6x1121	GY40	4	GY	WW	4.0	2.5	10.0	6_14	ZM00146008	ZM00149291	CKL09002
72	6x1015	ASI33	4	ASI	WW	2.6	6.2	14.0	4_16	ZM00151306	ZM00149064	CZL04003
73	6x1122	ASI34-WS	4	ASI	WS	2.9	3.5	26.0	22_30	ZM00148614	ZM00147855	CKL09006
74	6x1117	GY41	4	GY	WW	4.0	8.7	30.0	26_34	ZM00147680	ZM00146138	CKL09007
75	6x1116	ASI35	4	ASI	WW	2.7	6.2	34.0	30_36	ZM00150907	ZM00146576	CKL09007
76	6x1121	GY42	4	GY	WW	3.6	1.2	40.0	38_44	ZM00146375	ZM00149641	CKL09002
77	6x1028	GY43	4	GY	WW	3.6	8.0	52.0	48_54	ZM00146087	ZM00147968	VL062645
78	6x1028	ASI36	4	ASI	WW	8.7	17.3	54.0	52_56	ZM00147968	ZM00145459	VL062645
79	6x1120	ASI37-WS	4	ASI	WS	4.0	6.5	98.0	96_102	ZM00147666	ZM00149771	CKL09001
80	6x1018	GY44	4	GY	WW	3.1	4.8	116.0	108_120	ZM00146341	ZM00146213	CZL99017
81	6x1020	ASI38-WS	4	ASI	WS	2.9	4.0	122.0	110_126	ZM00147170	ZM00146254	CZL0724
82	6x1121	GY45-WS	5	GY	WS	2.5	3.9	0.0	0_6	ZM00145506	ZM00148602	CKL09002
83	6x1116	ASI40	5	ASI	WW	2.6	2.8	6.0	4_8	ZM00147505	ZM00149114	CML395
84	6x1028	GY46	5	GY	WW	2.8	3.5	12.0	0_30	ZM00146756	ZM00145442	CZL074
85	6x1019	GY47	5	GY	WW	4.6	13.0	24.0	18_30	ZM00146527	ZM00151505	CZL0719
86	6x1118	GY48	5	GY	WW	2.5	7.6	28.0	26_30	ZM00147053	ZM00145478	CML444
87	6x1120	GY49	5	GY	WW	6.0	11.4	30.0	26_36	ZM00150572	ZM00151317	CML395
88	6x1116	GY50	5	GY	WW	2.7	1.8	32.0	30_36	ZM00146744	ZM00148731	CKL09007
89	6x1121	GY51	5	GY	WW	3.9	5.3	42.0	38_44	ZM00147756	ZM00146035	CKL09002
90	6x1015	GY52	5	GY	WW	2.5	3.0	52.0	48_56	ZM00145731	ZM00149781	CZL00009
91	6x1017	GY53	5	GY	WW	2.7	8.7	52.0	50_58	ZM00146793	ZM00147438	CZL00009
92	6x1021	GY54	5	GY	WW	4.9	9.9	60.0	58_62	ZM00145549	ZM00145659	CZL0723
93	6x1016	GY55	5	GY	WW	3.0	9.4	70.0	62_76	ZM00151343	ZM00151129	CZL00009
94	6x1008	GY56	5	GY	WW	3.3	6.1	74.0	68_78	ZM00148569	ZM00146035	CZL00009
95	6x1023	ASI41	5	ASI	WW	4.7	7.7	82.0	78_88	ZM00145873	ZM00145478	CZL0618
96	6x1021	GY57-WS	5	GY	WS	3.5	3.4	110.0	104_114	ZM00145680	ZM00151606	CZL0723
97	6x1015	GY58	5	GY	WW	3.1	4.2	118.0	112_124	ZM00148731	ZM00145341	CZL00009
98	6x1019	ASI42	5	ASI	WW	3.5	7.5	118.0	116_122	ZM00146069	ZM00147337	CZL04008
99	6x1016	GY59	5	GY	WW	2.8	7.6	126.0	122_144	ZM00147212	ZM00145680	CZL00009
100	6x1016	ASI43-WS	5	ASI	WS	4.3	1.8	128.0	124_132	ZM00147212	ZM00145728	CZL00009
101	6x1016	ASI44	5	ASI	WW	7.6	4.7	128.0	122_144	ZM00147212	ZM00145728	CZL00009
102	6x1018	GY60-WS	5	GY	WS	3.5	7.7	142.0	132_162	ZM00146744	ZM00145728	CML505
103	6x1008	GY61	5	GY	WW	3.5	5.5	164.0	158_164	ZM00145341	ZM00147618	CZL00009
104	6x1116	GY62-WS	6	GY	WS	3.1	2.4	0.0	0_2	ZM00150684	ZM00146199	CKL09007
105	6x1120	ASI53	6	ASI	WW	2.9	2.8	0.0	0_12	ZM00148148	ZM00151005	CKL09008
106	6x1015	ASI45	6	ASI	WW	3.4	7.8	2.0	0_10	ZM00146808	ZM00149379	CZL04003

107	6x1019	GY63	6	GY	WW	3.2	3.1	6.0	0_8	ZM00150684	ZM00150913	CZL04008
108	6x1122	ASI46	6	ASI	WW	3.7	6.0	16.0	10_20	ZM00146092	ZM00145971	CKL09006
109	6x1028	GY64	6	GY	WW	4.2	5.9	40.0	34_46	ZM00145792	ZM00148356	CZL074
110	6x1122	ASI47	6	ASI	WW	3.3	10.8	44.0	38_48	ZM00147376	ZM00145762	CZL03011
111	6x1121	GY65	6	GY	WW	5.2	14.8	46.0	44_46	ZM00145855	ZM00149656	CKL09002
112	6x1016	GY66	6	GY	WW	2.7	5.2	58.0	50_62	ZM00145871	ZM00145637	CZL00009
113	6x1120	ASI52	6	ASI	WW	3.4	6.1	72.0	64_78	ZM00150166	ZM00150103	CKL09008
114	6x1021	GY67	6	GY	WW	6.6	18.0	80.0	74_84	ZM00145762	ZM00149548	CZL0719
115	6x1024	ASI48	6	ASI	WW	4.5	2.4	82.0	80_86	ZM00145762	ZM00150976	CZL02001
116	6x1024	GY68	6	GY	WW	3.9	8.6	86.0	82_102	ZM00150976	ZM00147585	VL062590
117	6x1023	GY69	6	GY	WW	5.3	8.3	90.0	84_94	ZM00151524	ZM00145320	CZL0618
118	6x1021	GY70-WS	6	GY	WS	4.3	6.3	94.0	88_98	ZM00145828	ZM00148890	CZL0719
119	6x1023	ASI49-WS	6	ASI	WS	3.0	7.6	98.0	94_106	ZM00145320	ZM00145828	CZL0618
120	6x1023	ASI50	6	ASI	WW	5.7	16.2	98.0	94_106	ZM00145320	ZM00145828	CZL0618
121	6x1021	ASI51-WS	6	ASI	WS	2.9	1.5	116.0	112_122	ZM00150103	ZM00147479	CZL0723
122	6x1015	ASI54	7	ASI	WW	4.7	10.6	10.0	6_14	ZM00148038	ZM00146706	CZL00009
123	6x1023	GY71	7	GY	WW	8.0	12.4	18.0	10_26	ZM00146941	ZM00146139	VL062655
124	6x1021	GY72	7	GY	WW	3.8	8.3	32.0	22_34	ZM00150786	ZM00145934	CZL0719
125	6x1024	ASI55	7	ASI	WW	2.6	5.7	32.0	20_42	ZM00146941	ZM00149691	CZL02001
126	6x1028	ASI56	7	ASI	WW	3.9	2.2	34.0	32_36	ZM00149389	ZM00148038	CZL074
127	6x1021	GY73-WS	7	GY	WS	2.7	5.0	36.0	26_48	ZM00145934	ZM00147097	CZL0719
128	6x1017	ASI57	7	ASI	WW	5.3	12.3	42.0	36_48	ZM00149150	ZM00145887	CZL00009
129	6x1008	GY74-WS	7	GY	WS	2.7	4.8	46.0	44_50	ZM00151492	ZM00148038	CZL00009
130	6x1008	ASI58-WS	7	ASI	WS	4.3	9.0	46.0	44_50	ZM00151492	ZM00148038	CZL00009
131	6x1008	ASI59	7	ASI	WW	3.5	7.4	50.0	48_54	ZM00145921	ZM00146706	CZL00009
132	6x1118	GY75	7	GY	WW	2.7	4.1	52.0	48_52	ZM00147114	ZM00148616	CML444
133	6x1120	GY76	7	GY	WW	2.7	6.2	58.0	42_60	ZM00145961	ZM00149261	CML395
134	6x1017	ASI60-WS	7	ASI	WS	4.8	8.2	60.0	56_68	ZM00149216	ZM00150057	CZL00009
135	6x1021	GY77-WS	7	GY	WS	3.4	7.4	68.0	60_78	ZM00147512	ZM00147408	CZL0723
136	6x1016	GY78	7	GY	WW	3.0	5.4	70.0	62_80	ZM00146706	ZM00147512	CZL99017
137	6x1020	GY79-WS	7	GY	WS	2.9	1.9	80.0	74_90	ZM00148075	ZM00149897	CZL0724
138	6x1020	ASI61-WS	7	ASI	WS	3.8	6.9	90.0	82_94	ZM00149897	ZM00146081	CZL0724
139	6x1120	ASI62-WS	7	ASI	WS	3.6	4.2	102.0	100_106	ZM00146668	ZM00148616	CML395
140	6x1117	ASI63-WS	8	ASI	WS	2.6	2.2	14.0	12_16	ZM00150790	ZM00151627	CKL09007
141	6x1021	GY80-WS	8	GY	WS	5.3	7.8	28.0	22_32	ZM00149771	ZM00150597	CZL0723
142	6x1121	GY81	8	GY	WW	8.8	17.9	28.0	22_40	ZM00150299	ZM00146174	CZL03011
143	6x1023	ASI64-WS	8	ASI	WS	3.4	4.3	40.0	24_48	ZM00146129	ZM00149199	CZL0618
144	6x1020	ASI65	8	ASI	WW	3.4	3.5	42.0	38_48	ZM00149380	ZM00146719	CZL0723
145	6x1020	ASI66-WS	8	ASI	WS	2.7	5.8	46.0	40_48	ZM00149380	ZM00146719	CZL0723
146	6x1117	ASI67-WS	8	ASI	WS	4.3	12.0	46.0	44_50	ZM00150059	ZM00146970	CKL09007
147	6x1028	GY82	8	GY	WW	6.3	13.6	50.0	42_52	ZM00150463	ZM00147090	CZL074
148	6x1015	GY83	8	GY	WW	4.3	8.4	56.0	52_58	ZM00150765	ZM00147431	CZL00009
149	6x1020	GY84-WS	8	GY	WS	2.6	4.2	58.0	54_64	ZM00146174	ZM00149211	CZL0723
150	6x1121	ASI68-WS	8	ASI	WS	3.2	9.2	60.0	50_68	ZM00151209	ZM00150573	CKL09002
151	6x1024	GY85	8	GY	WW	5.7	17.5	66.0	62_68	ZM00149832	ZM00147803	CZL02001
152	6x1028	GY86	8	GY	WW	2.6	4.8	70.0	64_70	ZM00147637	ZM00150597	VL062645
153	6x1120	ASI70	8	ASI	WW	2.9	8.2	82.0	68_86	ZM00146174	ZM00147431	CKL09008
154	6x1018	GY87	8	GY	WW	5.4	7.6	94.0	88_100	ZM00149841	ZM00147637	CZL99017
155	6x1020	ASI69-WS	8	ASI	WS	3.3	7.8	104.0	98_108	ZM00146654	ZM00146498	CZL0724
156	6x1115	GY88	8	GY	WW	3.7	2.7	112.0	102_116	ZM00146654	ZM00150573	CKL09004
157	6x1018	GY89	8	GY	WW	2.7	3.6	124.0	116_136	ZM00147620	ZM00150402	CML505
158	6x1024	GY90	9	GY	WW	2.7	5.5	4.0	0_8	ZM00148595	ZM00146298	CZL02001
159	6x1024	ASI71	9	ASI	WW	3.6	4.3	4.0	0_8	ZM00148595	ZM00146298	CZL02001
160	6x1117	ASI72-WS	9	ASI	WS	2.8	2.5	12.0	4_20	ZM00146298	ZM00149113	CML444
161	6x1017	GY91	9	GY	WW	3.3	8.0	24.0	18_32	ZM00145439	ZM00150601	CZL00009
162	6x1015	ASI73	9	ASI	WW	4.6	1.5	28.0	26_30	ZM00150601	ZM00151266	CZL04003
163	6x1116	ASI74	9	ASI	WW	2.6	1.7	28.0	26_30	ZM00151035	ZM00145408	CML395

164	6x1021	GY92-WS	9	GY	WS	3.7	3.3	30.0	28_34	ZM00146298	ZM00150248	CZL0719
165	6x1024	ASI76	9	ASI	WW	2.8	2.5	36.0	18_42	ZM00151468	ZM00148685	VL062590
166	6x1121	ASI75-WS	9	ASI	WS	3.4	3.7	36.0	30_42	ZM00150428	ZM00151231	CZL03011
167	6x1028	GY93	9	GY	WW	3.7	5.2	48.0	40_58	ZM00146800	ZM00151035	CZL074
168	6x1021	ASI77	9	ASI	WW	2.9	4.2	48.0	38_52	ZM00150703	ZM00148817	CZL0719
169	6x1023	ASI78	9	ASI	WW	2.8	5.0	56.0	44_66	ZM00151468	ZM00148082	CZL0618
170	6x1028	ASI79-WS	9	ASI	WS	3.9	2.7	62.0	58_68	ZM00149322	ZM00151224	VL062645
171	6x1017	GY94	9	GY	WW	5.8	13.6	70.0	64_76	ZM00147493	ZM00145411	CML539
172	6x1020	GY95	9	GY	WW	2.6	1.2	78.0	70_84	ZM00151559	ZM00145408	CZL0723
173	6x1015	GY96	9	GY	WW	6.1	9.0	80.0	72_84	ZM00150625	ZM00147953	CZL04003
174	6x1020	ASI80-WS	9	ASI	WS	3.4	6.1	90.0	86_96	ZM00145937	ZM00148777	CZL0723
175	6x1008	GY97	9	GY	WW	3.0	4.3	94.0	82_98	ZM00150625	ZM00150318	CML505
176	6x1122	GY99	10	GY	WW	2.9	1.5	0.0	0_4	ZM00148757	ZM00147067	CKL09006
177	6x1116	GY100	10	GY	WW	4.6	18.2	12.0	10_14	ZM00146514	ZM00151364	CML395
178	6x1028	ASI81-WS	10	ASI	WS	3.5	3.2	12.0	8_16	ZM00146726	ZM00145484	CZL074
179	6x1116	ASI82	10	ASI	WW	4.7	6.9	12.0	10_14	ZM00146514	ZM00151364	CML395
180	6x1116	GY101	10	GY	WW	9.5	19.1	24.0	22_28	ZM00146973	ZM00146628	CKL09007
181	6x1116	ASI83	10	ASI	WW	5.5	9.5	24.0	22_28	ZM00146973	ZM00146628	CKL09007
182	6x1020	GY102	10	GY	WW	6.0	4.6	26.0	22_28	ZM00147067	ZM00146248	CZL0724
183	6x1120	ASI84-WS	10	ASI	WS	4.0	6.5	46.0	44_52	ZM00146500	ZM00149827	CKL09008
