# Supplementary material for: Meta-analyses of QTL for grain yield and anthesis silking interval in 18 maize populations evaluated under water-stressed and well-watered environments
Source: BMC Genomics. 2013 May 10;14:313. doi: 10.1186/1471-2164-14-313 (PMC3751468; doi:10.1186/1471-2164-14-313)

**Additional file 2:** The projected positions of the 183 QTL for grain yield (GY) and anthesis-silking interval (ASI) using BioMercator version 3.0. The number at the bottom of each bar refers to the chromosome number. To minimize overlaps in font size of the markers, only part of the SNPs in the consensus maps were automatically displayed.


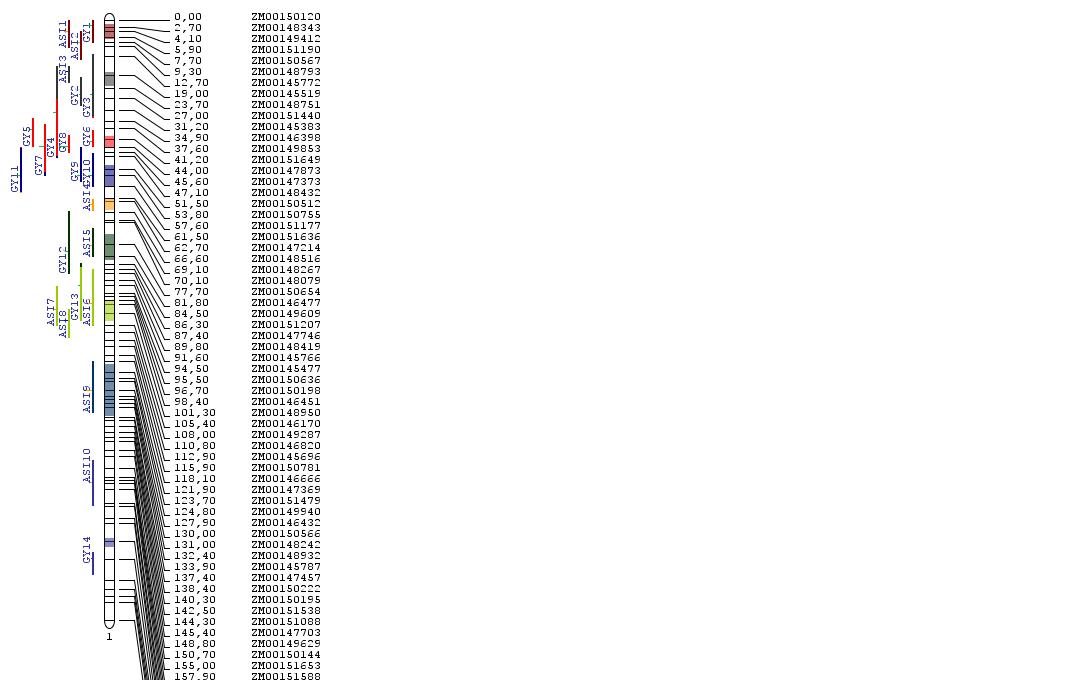

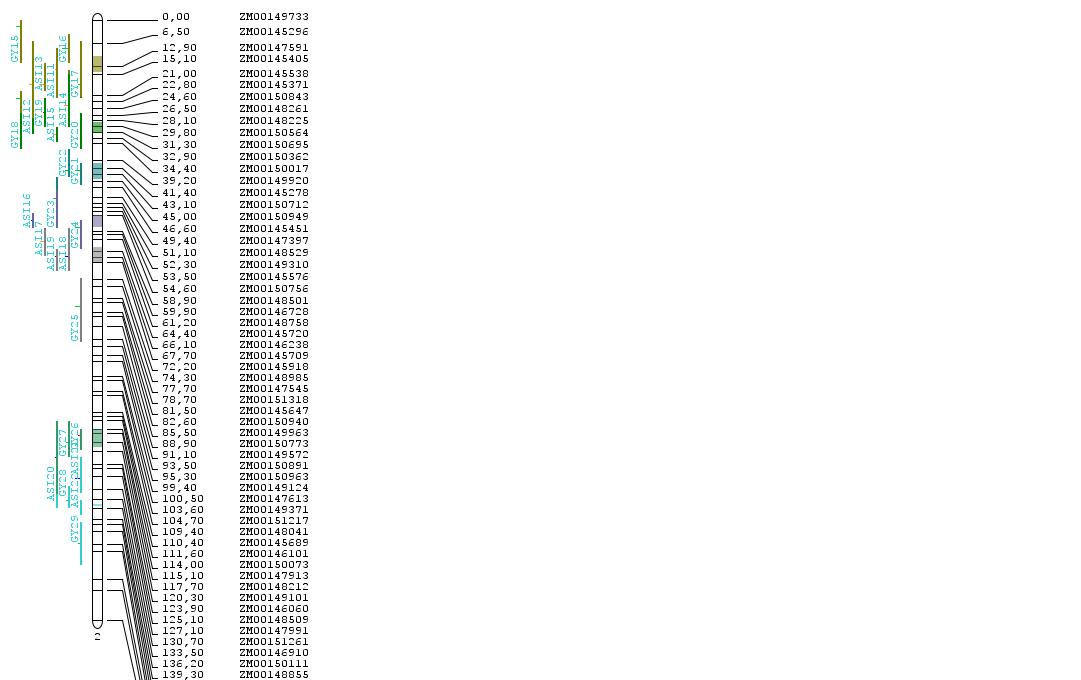


###
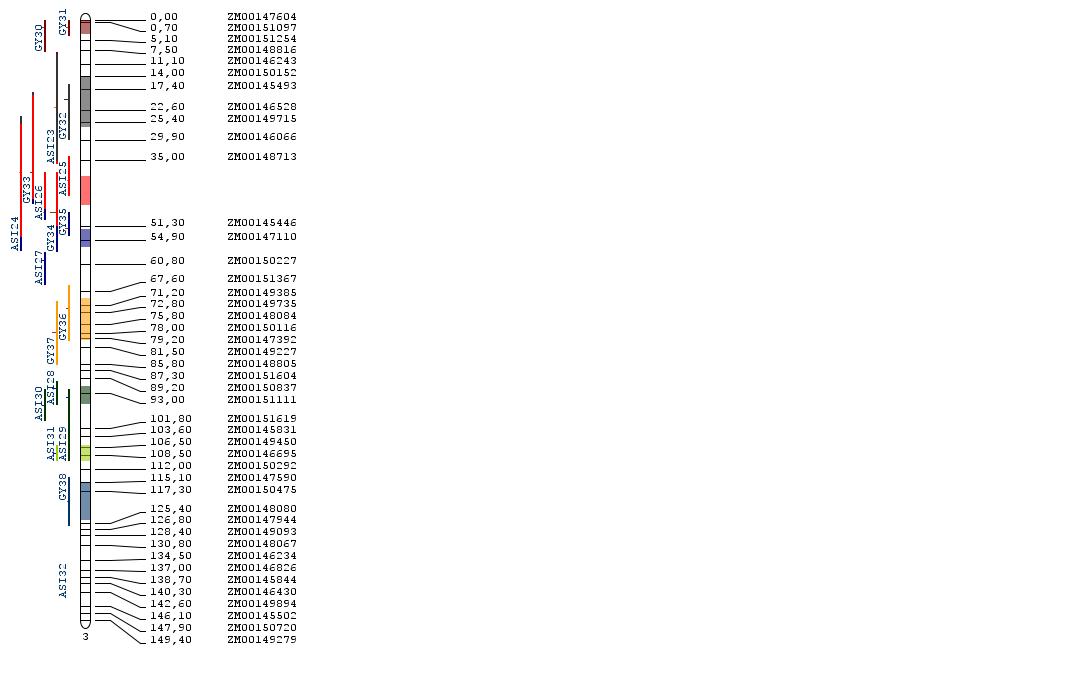

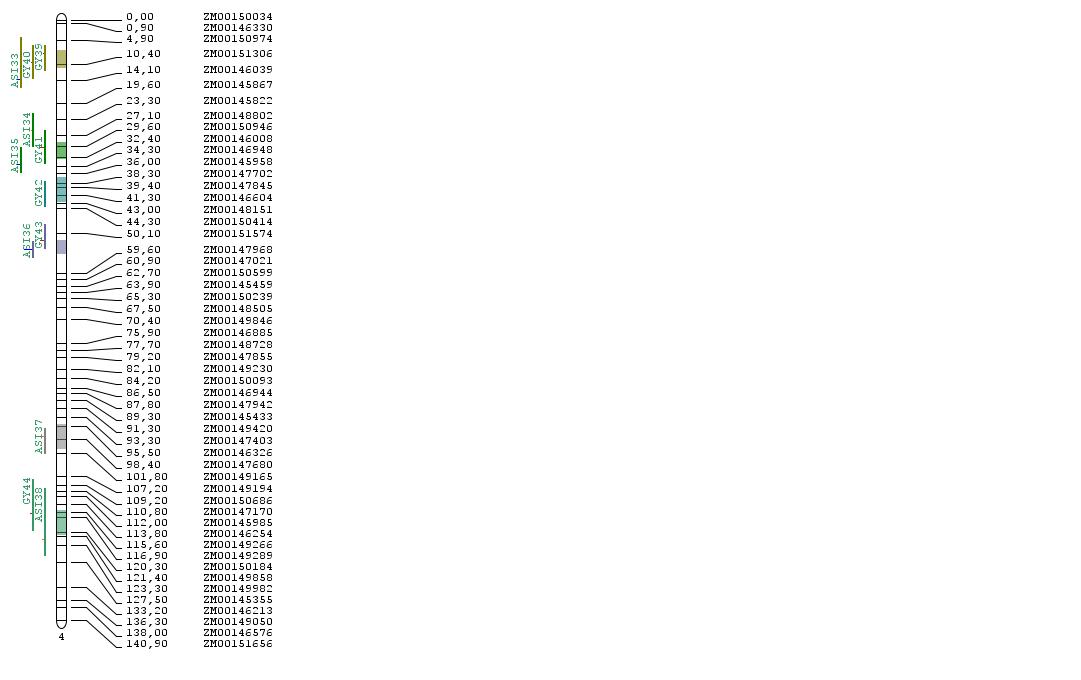


###
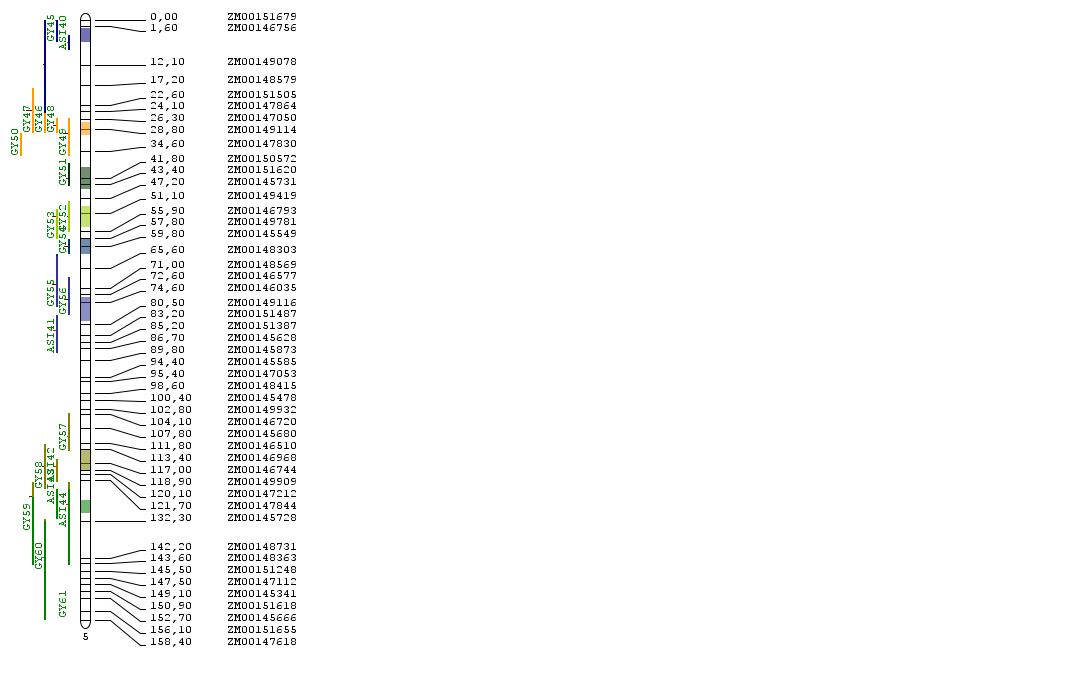

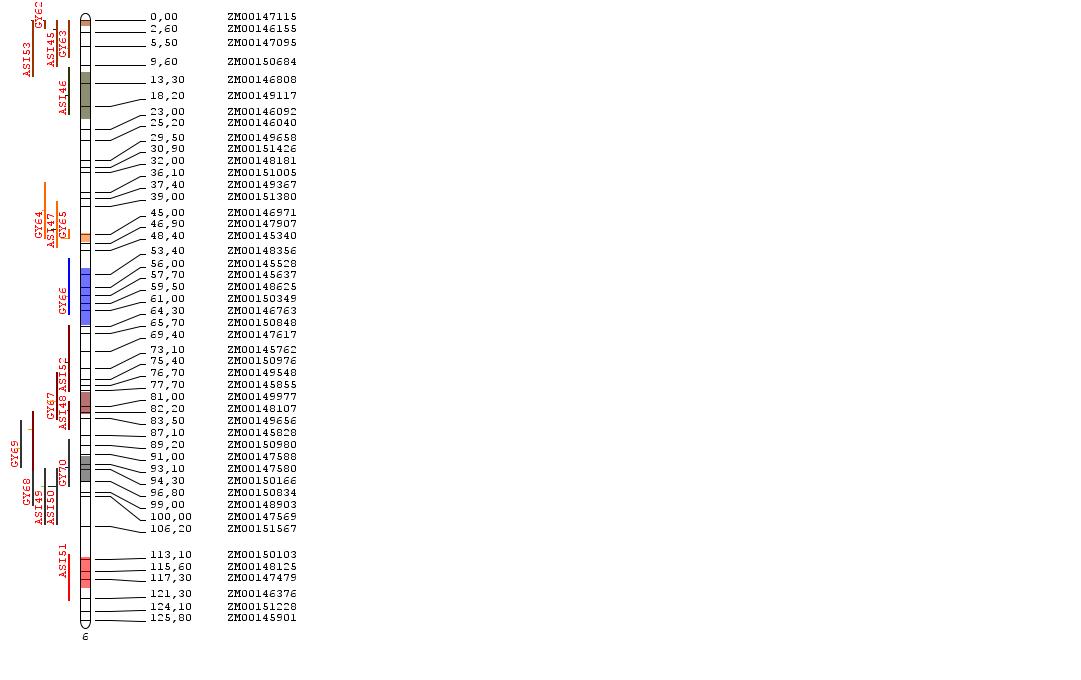


###
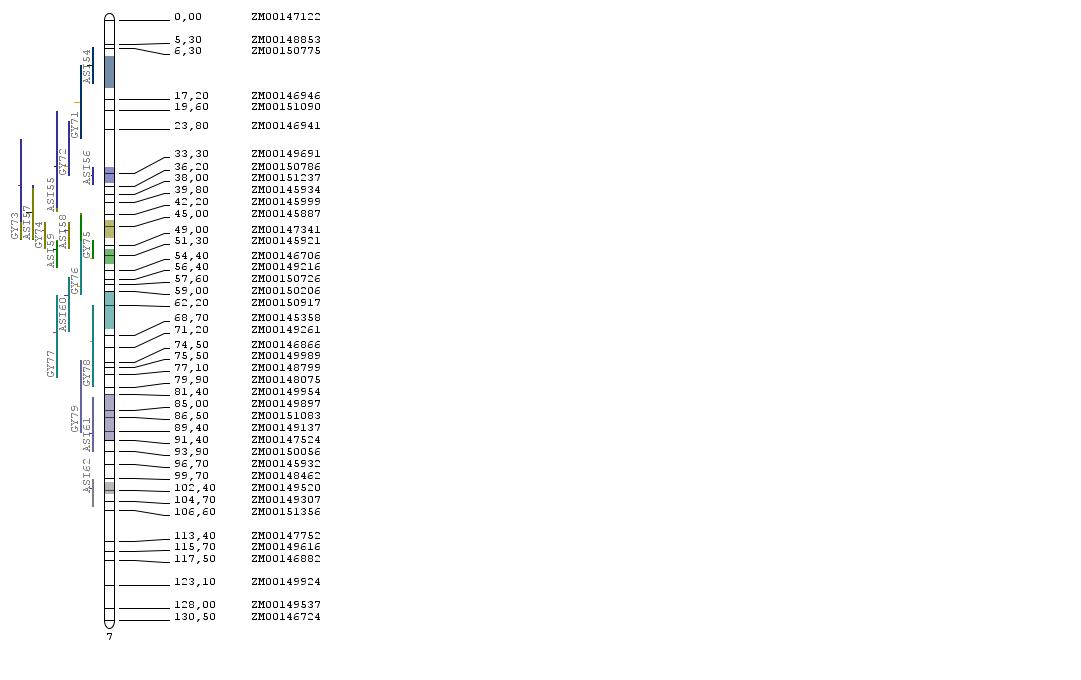

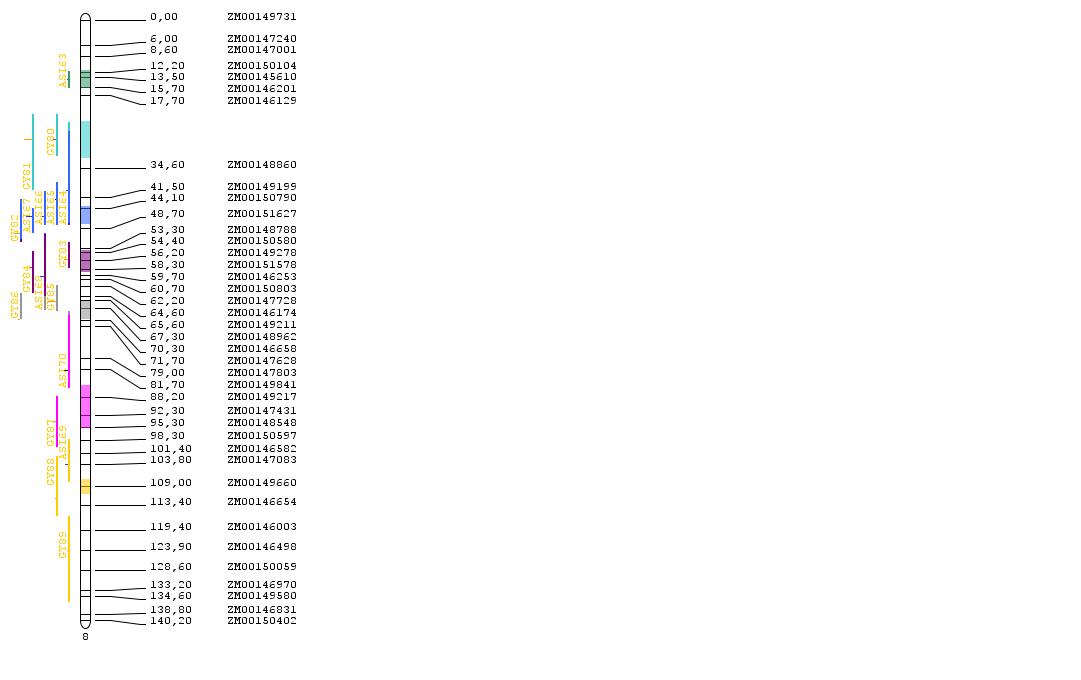


###
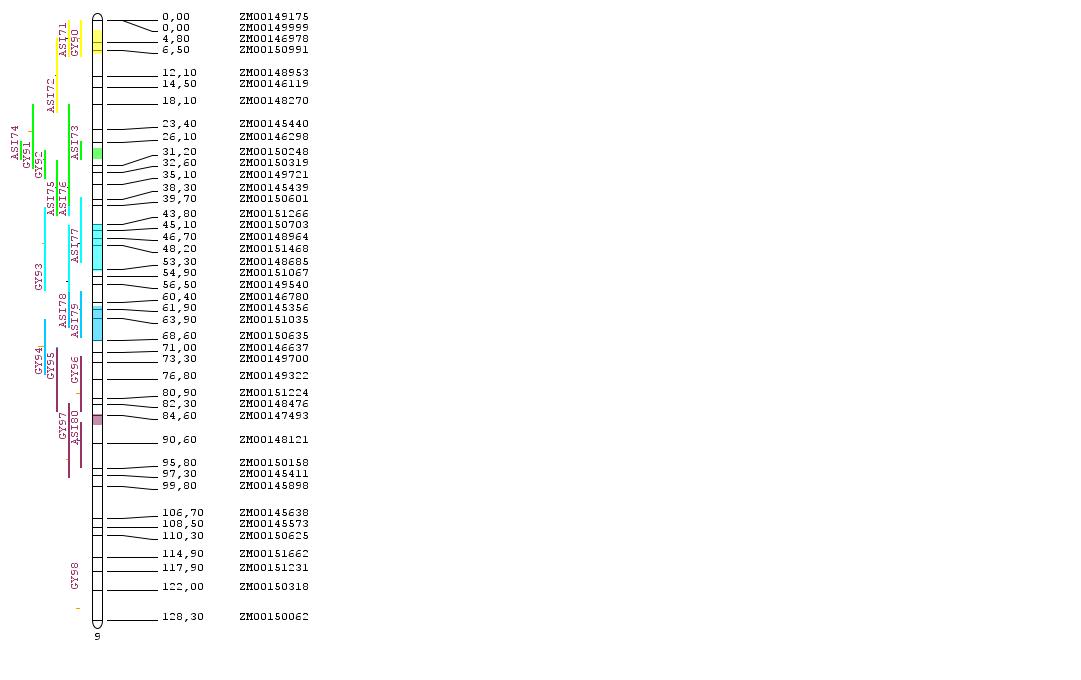

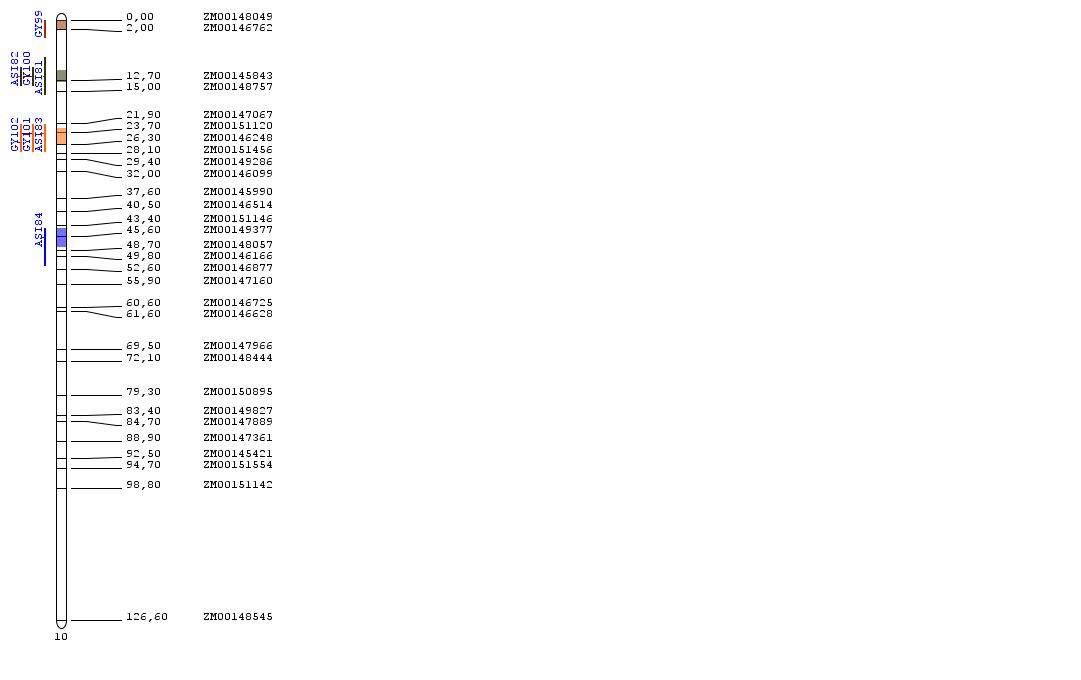

Supplement: Additional file 2 — Summary of the projected position of the 183 QTL for grain yield (GY) and anthesis-silking interval (ASI) using BioMercator version 3.0. [file 1471-2164-14-313-S2.docx]
