# Supplementary material for: Meta-analyses of QTL for grain yield and anthesis silking interval in 18 maize populations evaluated under water-stressed and well-watered environments
Source: BMC Genomics. 2013 May 10;14:313. doi: 10.1186/1471-2164-14-313 (PMC3751468; doi:10.1186/1471-2164-14-313)
Supplement: Additional file 4 — Summarizes the model selection criteria in the meta-analyses. [file 1471-2164-14-313-S4.docx]

**Additional file 4**: Summary of the model selection criteria in the meta-analyses. For each model within a chromosome, the value that best predicted the number of meta QTLs (K) is shaded and the selected models are shown in bold face.

| Chromosome | K | AIC | AICc | AIC3 | BIC | AWE |
| --- | --- | --- | --- | --- | --- | --- |
| 1 | 1 | 4248.0 | 4248.2 | 4249.0 | 4249.2 | 4255.4 |
| 1 | 2 | 1722.9 | 1724.1 | 1725.9 | 1726.4 | 1711.1 |
| 1 | 3 | 825.0 | 828.3 | 830.0 | 830.8 | 811.8 |
| 1 | 4 | 539.6 | 546.6 | 546.6 | 547.9 | 528.5 |
| 1 | 5 | 410.4 | 423.3 | 419.4 | 421.0 | 406.3 |
| 1 | 6 | 346.7 | 368.7 | 357.7 | 359.7 | 348.5 |
| 1 | 7 | 318.7 | 355.1 | 331.7 | 334.0 | 327.3 |
| 1 | 8 | 297.5 | 357.5 | 312.5 | 315.2 | 315.5 |
| 1 | 9 | 284.8 | 386.8 | **301.8** | **304.8** | **312.8** |
| 1 | 10 | 283.6 | 473.6 | 302.6 | 306.0 | 323.1 |
| 1 | 24 | 266.3 | 266.3 | 290.3 | 294.6 | 321.7 |
| 2 | 1 | 8100.5 | 8100.6 | 8101.5 | 8101.8 | 8108.0 |
| 2 | 2 | 1955.5 | 1956.5 | 1958.5 | 1959.4 | 1947.3 |
| 2 | 3 | 766.9 | 769.7 | 771.9 | 773.3 | 746.4 |
| 2 | 4 | 618.6 | 624.5 | 625.6 | 627.7 | 603.4 |
| 2 | 5 | 478.1 | 488.7 | 487.1 | 489.7 | 469.6 |
| 2 | 6 | 353.9 | 371.5 | 364.9 | 368.1 | 347.5 |
| 2 | 7 | 303.7 | **331.7** | **316.7** | **320.5** | **305.7** |
| 2 | 8 | 301.8 | 345.4 | 316.8 | 321.2 | 314.6 |
| 2 | 9 | 286.3 | 354.3 | 303.3 | 308.4 | 307.6 |
| 2 | 10 | 286.1 | 394.6 | 305.1 | 310.7 | 320.1 |
| 2 | 27 | 294.4 | 294.4 | 321.4 | 329.4 | 357.0 |
| 3 | 1 | 2387.9 | 2388.2 | 2388.9 | 2388.8 | 2394.7 |
| 3 | 2 | 1475.7 | 1477.4 | 1478.7 | 1478.4 | 1473.0 |
| 3 | 3 | 385.6 | 390.6 | 390.6 | 390.1 | 383.2 |
| 3 | 4 | 267.6 | 278.8 | 274.6 | 273.8 | 268.7 |
| 3 | 5 | 232.0 | 254.5 | 241.0 | 240.0 | 238.9 |
| 3 | 6 | 212.2 | 256.2 | 223.2 | 222.0 | 226.8 |
| 3 | 7 | 197.2 | 288.2 | 210.2 | 208.8 | 221.5 |
| 3 | 8 | 192.4 | 432.4 | 207.4 | 205.8 | 226.2 |
| 3 | 9 | 185.4 | 185.4 | 202.4 | 200.5 | 230.0 |
| 3 | 10 | 189.3 | 189.3 | 208.3 | 206.2 | 246.6 |
| 3 | 18 | 189.3 | 189.3 | 207.3 | 205.4 | 229.9 |
| 4 | 1 | 3411.7 | 3412.1 | 3412.7 | 3412.2 | 3417.6 |
| 4 | 2 | 1117.7 | 1120.7 | 1120.7 | 1119.1 | 1122.1 |
| 4 | 3 | 381.7 | 391.7 | 386.7 | 384.1 | 385.7 |
| 4 | 4 | 173.5 | 201.5 | 180.5 | 176.9 | 182.7 |
| 4 | 5 | 157.5 | 247.5 | 166.5 | 161.9 | 174.2 |
| 4 | 6 | 116.6 | 116.6 | **127.6** | **122.0** | **141.4** |
| 4 | 7 | 116.0 | 116.0 | 129.0 | 122.3 | 149.7 |
| 4 | 8 | 120.0 | 120.0 | 135.0 | 127.3 | 165.2 |
| 4 | 9 | 124.0 | 124.0 | 141.0 | 132.2 | 181.2 |
| 4 | 10 | 128.0 | 128.0 | 147.0 | 137.2 | 197.1 |
| 4 | 12 | 116.1 | 116.1 | 128.1 | 121.9 | 133.6 |
| 5 | 1 | 3826.2 | 3826.4 | 3827.2 | 3827.2 | 3833.3 |
| 5 | 2 | 3131.6 | 3133.0 | 3134.6 | 3134.7 | 3123.8 |
| 5 | 3 | 928.1 | 932.1 | 933.1 | 933.3 | 917.4 |
| 5 | 4 | 407.1 | 415.7 | 414.1 | 414.4 | 401.0 |
| 5 | 5 | 309.1 | 325.5 | 318.1 | 318.5 | 309.0 |
| 5 | 6 | 260.3 | 289.7 | 271.3 | 271.8 | 269.7 |
| 5 | 7 | 240.0 | 292.0 | 253.0 | 253.6 | 255.7 |
| 5 | 8 | 227.3 | 323.3 | **242.3** | **243.0** | **253.5** |
| 5 | 9 | 227.1 | 431.1 | 244.1 | 244.8 | 263.6 |
| 5 | 10 | 227.1 | 987.1 | 246.1 | 246.9 | 274.3 |
| 5 | 21 | 225.3 | 225.3 | 246.3 | 247.2 | 266.8 |
| 6 | 1 | 6172.9 | 6173.2 | 6173.9 | 6173.8 | 6179.7 |
| 6 | 2 | 809.3 | 811.0 | 812.3 | 811.9 | 807.4 |
| 6 | 3 | 363.2 | 368.2 | 368.2 | 367.7 | 359.8 |
| 6 | 4 | 253.1 | 264.3 | 260.1 | 259.3 | 251.0 |
| 6 | 5 | 212.7 | 235.2 | 221.7 | 220.7 | 220.4 |
| 6 | 6 | 205.6 | 249.6 | 216.6 | 215.4 | 222.5 |
| 6 | 7 | 178.5 | 269.5 | **191.5** | **190.1** | **203.9** |
| 6 | 8 | 178.5 | 418.5 | 193.5 | 191.9 | 214.4 |
| 6 | 9 | 180.1 | 180.1 | 197.1 | 195.3 | 226.2 |
| 6 | 10 | 183.3 | 183.3 | 202.3 | 200.2 | 241.3 |
| 6 | 18 | 184.2 | 184.2 | 202.2 | 200.2 | 220.8 |
| 7 | 1 | 2264.4 | 2264.7 | 2265.4 | 2265.3 | 2271.2 |
| 7 | 2 | 730.8 | 732.5 | 733.8 | 733.4 | 735.3 |
| 7 | 3 | 340.6 | 345.6 | 345.6 | 345.0 | 339.0 |
| 7 | 4 | 234.3 | 245.5 | 241.3 | 240.5 | 239.1 |
| 7 | 5 | 197.1 | 219.6 | 206.1 | 205.1 | 206.6 |
| 7 | 6 | 184.6 | 228.6 | 195.6 | 194.4 | 202.3 |
| 7 | 7 | 176.0 | 267.0 | **189.0** | **187.6** | **201.0** |
| 7 | 8 | 180.0 | 420.0 | 195.0 | 193.4 | 218.5 |
| 7 | 9 | 182.7 | 182.7 | 199.7 | 197.9 | 233.2 |
| 7 | 10 | 186.6 | 186.6 | 205.6 | 203.5 | 250.6 |
| 7 | 18 | 187.4 | 187.4 | 205.4 | 203.4 | 226.7 |
| 8 | 1 | 2959.8 | 2960.1 | 2960.8 | 2960.7 | 2966.6 |
| 8 | 2 | 1208.9 | 1210.6 | 1211.9 | 1211.5 | 1204.5 |
| 8 | 3 | 435.6 | 440.6 | 440.6 | 440.0 | 434.3 |
| 8 | 4 | 256.2 | 267.4 | 263.2 | 262.4 | 257.8 |
| 8 | 5 | 225.0 | 247.5 | 234.0 | 233.0 | 231.0 |
| 8 | 6 | 200.1 | 244.1 | 211.1 | 209.8 | 216.1 |
| 8 | 7 | 187.9 | 278.9 | **200.9** | **199.4** | **212.2** |
| 8 | 8 | 190.9 | 430.9 | 205.9 | 204.3 | 226.8 |
| 8 | 9 | 189.8 | 189.8 | 206.8 | 204.9 | 237.5 |
| 8 | 10 | 192.9 | 192.9 | 211.9 | 209.8 | 252.2 |
| 8 | 18 | 191.4 | 191.4 | 209.4 | 207.4 | 226.2 |
| 9 | 1 | 3265.4 | 3265.7 | 3266.4 | 3266.4 | 3272.3 |
| 9 | 2 | 532.9 | 534.5 | 535.9 | 535.7 | 527.5 |
| 9 | 3 | 276.5 | 281.1 | 281.5 | 281.2 | 272.2 |
| 9 | 4 | 194.2 | 204.3 | 201.2 | 200.8 | 191.8 |
| 9 | 5 | **179.9** | **199.9** | **188.9** | **188.4** | **186.2** |
| 9 | 6 | 181.1 | 218.8 | 192.1 | 191.5 | 198.7 |
| 9 | 7 | 183.3 | 256.1 | 196.3 | 195.5 | 210.8 |
| 9 | 8 | 187.0 | 347.0 | 202.0 | 201.2 | 228.3 |
| 9 | 9 | 190.9 | 802.9 | 207.9 | 207.0 | 244.5 |
| 9 | 10 | 195.1 | 195.1 | 214.1 | 213.0 | 264.0 |
| 9 | 19 | 199.8 | 199.8 | 218.8 | 217.7 | 241.5 |
| 10 | 1 | 609.1 | 609.7 | 610.1 | 609.1 | 614.2 |
| 10 | 2 | 226.2 | 232.2 | 229.2 | 226.5 | 230.6 |
| 10 | 3 | 139.0 | 169.0 | 144.0 | 139.4 | 149.2 |
| 10 | 4 | **55.5** | **55.5** | **62.5** | **56.0** | **71.5** |
| 10 | 5 | 59.5 | 59.5 | 68.5 | 60.2 | 85.8 |
| 10 | 6 | 63.5 | 63.5 | 74.5 | 64.3 | 100.1 |
| 10 | 8 | 58.1 | 58.1 | 66.1 | 58.8 | 77.5 |
